# Enhancing the Efficiency and Flexibility of AutoMeKin: Integrating ORCA and Machine-Learning Potentials

**DOI:** 10.1021/acs.jctc.6c01565

**Published:** 2026-10-06

**Authors:** Omar Rodríguez-López, Emilio Martínez-Núñez, Berta Fernández, Saulo A. Vázquez

**Affiliations:** Departamento de Química Física, 16780 Universidade de Santiago de Compostela, Santiago de Compostela E-15782, Spain

## Abstract

Automated reaction discovery is often limited by the cost of high-level refinement of large reaction networks. Here, we extend AutoMeKin, an automated reaction-discovery program, by presenting a complete integration of ORCA 6 into its high-level refinement workflow, providing access to a broad range of electronic-structure methods through a package freely available to academic users. We also integrate the UMA and MACE-OMOL-0 machine-learning interatomic potentials and Δ-ML energy corrections, enabling automated refinement of reaction networks using conventional electronic-structure methods, foundation ML potentials, or ML-corrected quantum-mechanical approaches within a unified framework. The workflow is assessed for five systems, monoethanolamine, three cyclobutadiene-derived radicals, and *N*-[bis­(2H-triazol-4-yl)­methylidene]­hydroxylamine, spanning both in-distribution and challenging out-of-distribution chemistry. Comparisons with reference data show that UMA and MACE-OMOL-0 generally recover the relevant reaction mechanisms and provide energy profiles of comparable quality to DFT at substantially reduced cost. RRKM analyses further reveal that vibrational frequencies can significantly affect product population fractions, especially for loose transition states. These results show that the new implementation increases the accessibility and flexibility of AutoMeKin and further support the view that foundation ML potentials can serve as practical surrogates for DFT in automated reaction-network discovery.

## Graphical abstract

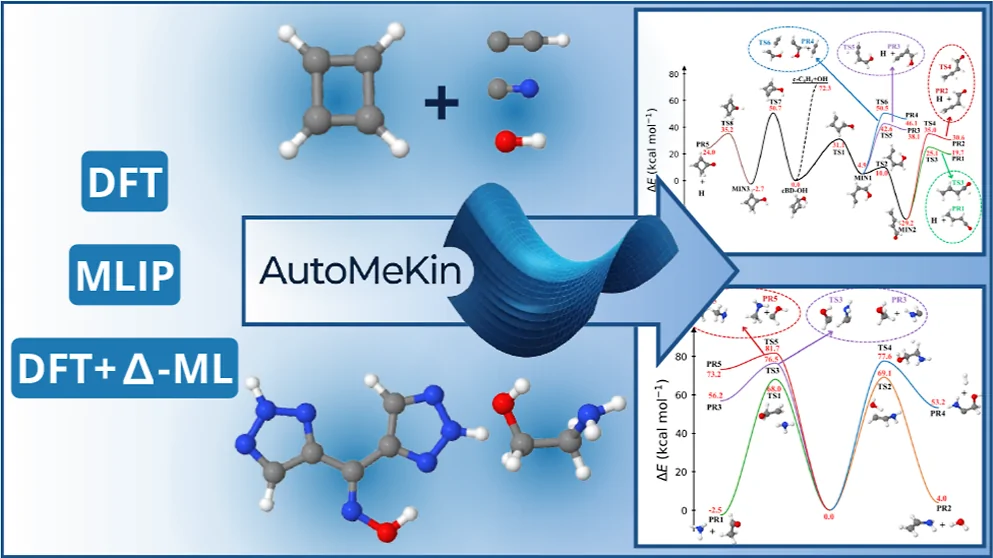

## 1 Introduction

Automated reaction discovery is transforming the investigation of chemical reactivity by enabling the exploration of large regions of chemical space without predefined mechanistic assumptions.^1,2^ Numerous methods and software packages have been developed for the autonomous exploration of reaction networks.^1,2^ Among them, AutoMeKin,^3–6^ developed in our laboratory, provides an open-source framework for the automated exploration of potential energy surfaces (PESs), identification of reaction pathways, and construction of reaction networks across a wide range of chemical systems. Since the release of AutoMeKin2021,^6^ the software has been applied to numerous problems that span combustion, atmospheric chemistry, astrochemistry, catalysis, mass spectrometry, and synthetic chemistry.^7^

To further enhance the flexibility of AutoMeKin and broaden its appeal to the computational chemistry community, the present work incorporates a comprehensive interface to ORCA 6,^8,9^ thereby expanding the repertoire of electronic-structure packages available within the program. This interface builds on the simpler ORCA implementation introduced in a recent work,^10^ where ORCA was used to perform selected electronic-structure calculations within a post-processing workflow associated with AutoMeKin. The new implementation provides direct access to ORCA, complementing the existing interfaces to Gaussian^11^ and Entos Qcore^12^ and giving users access to a broader range of electronic-structure methods and functionalities within a unified automated workflow. This addition expands the theoretical tools available to AutoMeKin users while maintaining the automated and general-purpose character of the software.

While the new ORCA interface considerably broadens the range of electronic-structure methods available to AutoMeKin users, the computational cost of high-level calculations remains a major challenge for automated reaction discovery. Accurate methods implemented in packages such as Gaussian and ORCA can provide reliable characterizations of PESs; however, their systematic use becomes impractical in exhaustive explorations where hundreds or thousands of stationary points and reaction pathways may be generated. A common strategy is therefore to perform the initial exploration with low-cost electronic-structure methods and then refine the resulting network at a higher level of theory. Although this multilevel approach substantially reduces the computational burden, the refinement stage often remains the dominant bottleneck, particularly when accurate barrier heights (BHs) and reaction energies (REs) are required.

Machine-learning (ML) methods have emerged as a promising route to bridge the gap between computational cost and accuracy. In our previous work,^10^ we developed ML corrections for several low- and mid-level electronic-structure methods, including PM6,^13^ HF/3-21G,^14^ and r^2^SCAN-3c,^15^ using a Δ-ML strategy trained on coupled-cluster reference energies. Implemented as a post-processing workflow associated with AutoMeKin, these models substantially improved the accuracy of the underlying quantum-mechanical methods, with the corrected r^2^SCAN-3c approach achieving near-coupled-cluster performance for BHs and REs.

The success of ML models, however, depends critically on the quality, diversity, and coverage of the training data. Important benchmarks for reaction energetics include the datasets developed by Green and co-workers,^16,17^ which contain approximately 12,000 gas-phase reactions involving molecules composed of H, C, N, and O atoms. These datasets provide density functional theory (DFT) geometries together with coupled-cluster reference energetics and have become valuable resources for the development and validation of ML approaches to chemical reactivity; they also served as the training data for the Δ-ML models developed in ref 10, including the *r* ^2^SCAN-3c ML-CC approach employed in the present work. Nevertheless, their chemical scope remains relatively limited compared with the diversity encountered in realistic reaction-network explorations.

Recently, machine-learning interatomic potentials (MLIPs) or foundation models trained on unprecedentedly large and chemically diverse datasets have begun to redefine the capabilities of ML interatomic potentials. The Open Molecules 2025 (OMol25) dataset^18^ contains more than 140 million quantum-chemical calculations spanning molecular systems with diverse compositions, charge states, spin multiplicities, conformations, and reactive configurations, making it one of the most chemically diverse training resources currently available. Built upon datasets such as OMol25 and related collections covering materials, catalysts, interfaces, and molecular crystals, the Universal Models for Atoms (UMA)^19^ have emerged as highly transferable foundation potentials capable of achieving near-DFT accuracy across a broad range of chemical domains. Similarly, the recently introduced MACE-OMOL-0 model^20^ extends the MACE framework^21^ through large-scale training on OMol25, providing another foundation model approach for molecular simulations.

The combination of automated reaction exploration and ML interatomic potentials represents a particularly natural pairing: the computational efficiency of ML potentials directly addresses the refinement bottleneck that limits the scalability of autonomous workflows, while the diversity of reaction networks generated by automated methods provides a stringent and practically relevant test bed for ML transferability. This synergy has begun to attract increasing attention. Young and co-workers^22^ demonstrated that hierarchical active-learning Gaussian Approximation Potential models, requiring only hundreds of quantum-chemical evaluations, can be integrated with the autodE automated reaction-discovery framework to study reactive dynamics in both gas and condensed phases, including S_N_2 substitutions and bifurcating Diels–Alder pathways. Ghorai and co-workers^23^ demonstrated that an iteratively trained Δ-learning neural network potential, built on top of a semiempirical baseline and progressively refined on SC-AFIR reaction path data, achieves DFT-level accuracy for the automated exploration of alkene polymerization and metallacycle formation networks. Independently, Kendall and Zádor^24^ benchmarked UMA within the KinBot automated kinetics workflow across 12 gas-phase systems relevant to combustion and atmospheric chemistry, finding that UMA reliably identifies expected reaction channels and that single-point DFT corrections at UMA-optimized geometries largely recover the remaining energy discrepancies.

In addition to the improved ORCA interface, the present work integrates UMA and MACE-OMOL-0 into AutoMeKin and evaluates them for the refinement of reaction networks, alongside the *r* ^2^SCAN-3c ML-CC model from ref 10, which serves as a complementary ML-based method. Our purpose is not to propose *r* ^2^SCAN-3c ML-CC as a practical alternative for AutoMeKin applications, as it is not computationally efficient compared with universal ML foundation models. Rather, we aim to assess the transferability of our Δ-ML corrections to systems that differ substantially from those represented in the training set. Moreover, the Δ-ML implementation in AutoMeKin provides a framework for incorporating ML-CC corrections into UMA or MACE-OMOL-0 models in future work. All approaches are benchmarked against DFT and coupled-cluster reference data for chemically diverse systems: monoethanolamine (MEA), three cyclobutadiene (cBD)-derived radicals (cBD-CN, cBD-CCH, and cBD–OH), and *N*-[bis­(2H-triazol-4-yl)­methylidene]­hydroxylamine (Tz2-HA, C_5_H_5_N_7_O). These systems were selected to probe both in-distribution and genuinely out-of-distribution (OOD) generalization across all methods evaluated. Notably, MEA occupies a doubly in-distribution position: its molecular formula is represented in the OMol25 dataset (with 3649 entries), used to train UMA and MACE-OMOL-0, and three MEA conformers are present in the Green datasets^16,17^ used to train the Δ-ML models. In contrast, to the best of our knowledge, the molecular formulas of the cyclobutadiene derivatives and Tz2-HA are absent from both training sets, making them genuinely OOD test cases.

The paper is organized as follows: Section 2 describes the updated AutoMeKin workflow following the integration of ORCA and ML models; Section 3 details the computational protocol and test systems; Section 4 reports and discusses the results; and Section 5 summarizes the main conclusions.

## 2 Updated AutoMeKin Workflow

The integration of ORCA^8,9^ follows the same workflow as that employed for Gaussian^11^ and has been described in previous works.^5,6^ The ML interatomic potentials were incorporated into the high-level refinement layer of AutoMeKin as alternatives to DFT calculations, covering geometry optimizations, energy evaluations, and minimum-energy-path (MEP) calculations connecting TSs and minima. This stage constitutes the second step of the workflow in which reaction networks generated during the PES exploration stage are refined using higher-level electronic-structure or data-driven methods. The methodologies described in this work have been implemented in AutoMeKin-X, a publicly available development version of AutoMeKin.^25^

Importantly, the present implementation of the Δ-ML framework extends that reported in ref 10 by fully integrating Δ-ML within the AutoMeKin workflow via user-level keywords, rather than relying on external post-processing scripts. These keywords, defined in the input control file, specify the Δ-ML protocol, baseline electronic-structure method, and the correction model and enable automatic correction of the energies of all stationary points stored in the corresponding SQLite tables and text files.

The modified AutoMeKin workflow is illustrated in Figure 1 and comprises two sequential stages: PES exploration and reaction network (RXN) refinement. For readers unfamiliar with AutoMeKin, we briefly summarize the PES exploration stage here; full algorithmic details can be found in refs 3–6 . In the first stage, an initial structure is propagated through molecular dynamics (MD)-based exploration algorithms that systematically sample configurational space at relatively low computational cost using a semiempirical electronic-structure method, as detailed in Section 3. Reactive events are generated from short, high-energy MD trajectories, with vibrational energy adaptively distributed among the normal modes to promote bond rearrangements on short time scales. Each trajectory is monitored using an adjacency matrix constructed from interatomic distances scaled by reference values based on covalent radii. A reactive event is identified when the closest neighbor of an atom switches from a covalently bonded partner to a non-bonded atom. Because a single transition state can involve multiple bond changes, reactive events occurring within a short adaptive time window are merged into a single event.^3^

**Figure 1:**
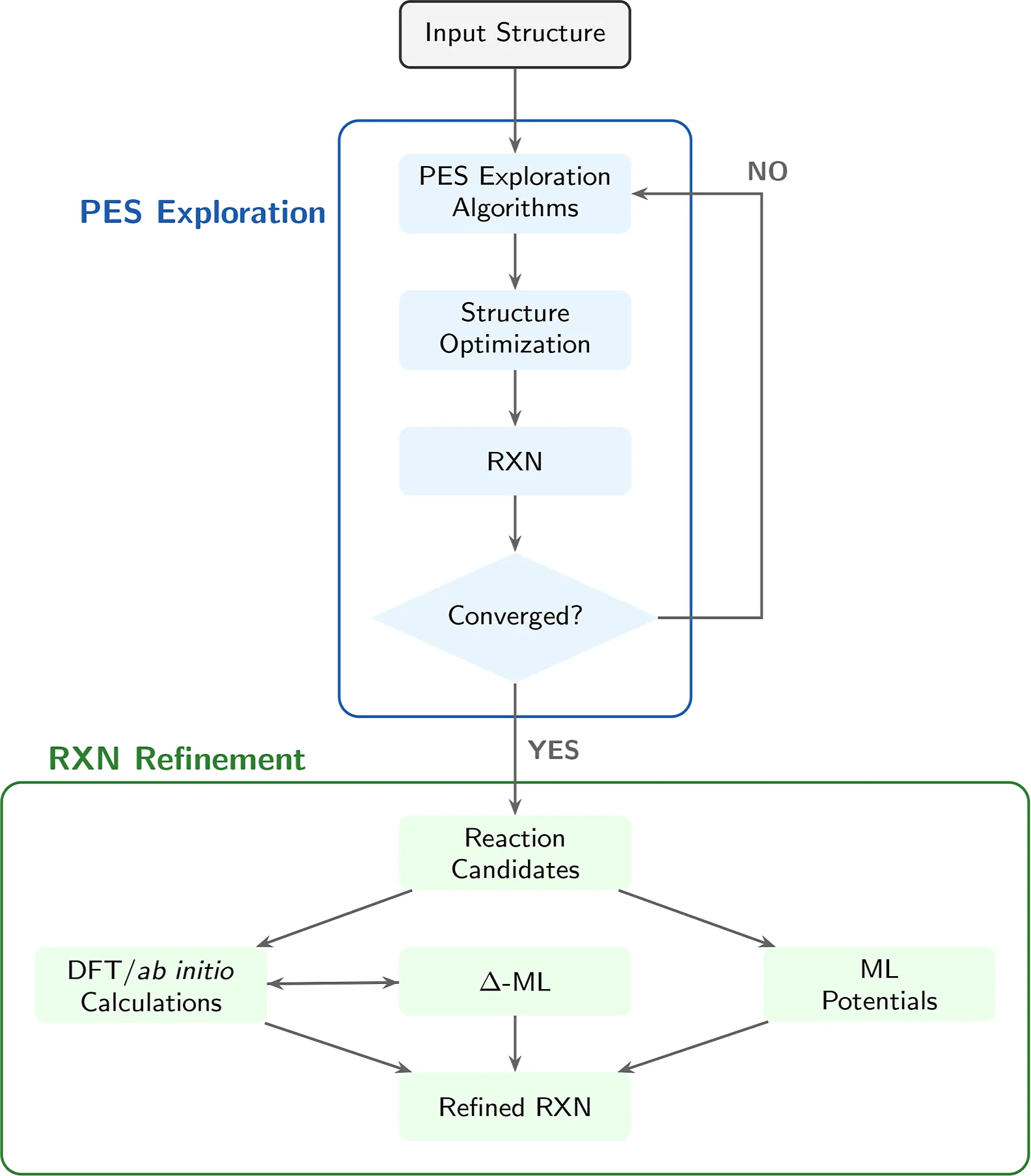
Modified AutoMeKin workflow. The PES exploration stage (blue) samples configurational space at a semiempirical level. Upon convergence, the resulting reaction candidates enter the RXN refinement stage (green), where three high-level pathways are available: DFT/ab initio calculations, Δ-ML, and MLIPs (UMA or MACE-OMOL-0).

The corresponding structures are then partially relaxed with the reactive atoms held fixed and subsequently optimized to identify the minima and first-order saddle points, yielding transition-state (TS) candidates. This default reactive event detection procedure is termed bbfs (bond breaking/formation search).^3^ The resulting TS candidates are connected to their corresponding reactant and product minima by low-level intrinsic reaction coordinate (IRC) calculations,^26^ thereby defining the elementary steps of a preliminary low-level RXN. Importantly, the initial MD simulation does not need to contain all reactants of the elementary reactions that are subsequently identified. AutoMeKin is designed primarily to explore the PES and generate structures resembling transition-state configurations through the bbfs procedure. Because the high-energy MD trajectories can explore a broad region of configurational space, they may encounter configurations corresponding to TSs of both unimolecular and bimolecular reactions even when the MD simulation is initiated from a single molecular structure. Thus, bimolecular elementary steps can be identified without specifying the number of molecules or the reactants involved a priori.

Each exploration iteration consists of multiple parallel tasks, with each typically involving 10 short MD trajectories. Convergence of the exploration stage is commonly assessed via high-temperature microkinetic modeling of the evolving reaction network, although the specific criterion is application-dependent;^27^ in the present work, convergence was instead defined by a fixed number of MD iterations, as described in Section 3. The PES exploration stage relied exclusively on the default MD/bbfs sampling; the rare-event acceleration method BXDE and the constraint-based ChemKnow approach, both available in AutoMeKin, were not employed.

In the RXN refinement stage, the transition-state structures identified during the low-level PES exploration are reoptimized using three alternative high-level approaches: conventional DFT/ab initio calculations, Δ-ML, and MLIPs. Here, a reaction candidate refers to a transition state, and all or a selected subset of the transition states identified during the low-level exploration can be subjected to high-level reoptimization. The successfully optimized TS structures are subsequently characterized by IRC calculations to determine the corresponding reaction paths and thereby identify the reactant and product minima associated with each elementary reaction. This procedure generates the refined high-level RXN, in which the connectivity and energetics of the elementary steps are determined consistently at the selected refinement level.

As described in ref 10, the Δ-ML framework combines a baseline electronic-structure method (PM6, HF/3-21G, or *r* ^2^SCAN-3c) with an ML correction trained to reproduce CC reference energies, yielding the PM6 ML-CC, HF/3-21G ML-CC, and *r* ^2^SCAN-3c ML-CC approaches, respectively. In this work, we employed the most accurate variant, *r* ^2^SCAN-3c ML-CC. The MLIP approach instead uses a machine-learning potential directly to provide energies and gradients during the refinement procedure, whereas conventional DFT/ab initio calculations perform the reoptimization and subsequent electronic-structure calculations explicitly at the selected level of theory. All three approaches yield refined RXNs with substantially improved structural and energetic accuracy relative to the initial semiempirical exploration. ORCA^8,9^ was used for all conventional high-level electronic-structure calculations in this study.

The present implementation extends the high-level refinement capabilities of AutoMeKin beyond those available in the original 2021 implementation (ref 6), which provided interfaces to Gaussian and Entos Qcore for DFT/ab initio calculations. The new implementation incorporates ORCA as well as MLIP and Δ-ML-based refinement, thereby providing a unified workflow in which the TS structures generated during the low-level exploration can be reoptimized and the corresponding reaction pathways and reaction networks refined using these alternative levels of theory.

Two MLIPs serve as backends in the present implementation: UMA^19^ and MACE-OMOL-0.^20^ UMA is a universal interatomic potential trained on chemically diverse datasets for broad transferability; specifically, the uma-m-1p1 model from the UMA family was employed in the present work. MACE-OMOL-0 is trained on OMol25 within the MACE equivariant message-passing framework, which uses higher order geometric representations to improve accuracy in complex bonding environments. In the present implementation, both potentials replace DFT in geometry optimization, TS refinement, and MEP construction, interfaced through the Atomistic Simulation Environment (ASE),^28^ which employs Sella^29^ as a saddle-point optimizer. Although DFT geometry optimizations and MEP calculations employ by default the native optimizers of the corresponding quantum chemistry program (Gaussian16 ^11^ or ORCA^8,9^ ), it is also possible to route these calculations through ASE using Sella as the optimizer. This shared interface ensures that DFT- and ML-based refinements employ identical optimization algorithms, facilitating direct comparison of the resulting reaction networks.

The above unified ML-based refinement strategy substantially reduces the computational cost of the second stage relative to conventional DFT while preserving full compatibility with standard electronic-structure workflows and allowing seamless switching between refinement backends.

## 3 Computational Protocol and Test Systems

### 3.1 Low-Level Reaction-Network Generation

The low-level reaction networks are generated following the PES Exploration stage described in Section 2, using the same AutoMeKin molecular dynamics (MD)-based exploration framework. Starting from the input structure, high-temperature MD simulations are performed to systematically sample the configurational space by using a semiempirical electronic-structure method.

Five test systems were investigated: MEA, three cyclobutadiene-derived radicals (cBD-X, X = CN, CCH, and OH), and Tz2-HA. The systems were selected to span a range of chemical complexity and to probe both in-distribution and OOD generalization of the ML potentials, as discussed in Section 1. For each system, the MD-based exploration procedure was executed for a fixed number of AutoMeKin iterations, chosen to ensure a sufficient number of reaction candidates in the resulting RXNs while accounting for system size. Specifically, the number of iterations was reduced for the largest system (Tz2-HA) to avoid generating an excessively large reaction network that would render the subsequent high-level refinement computationally intractable. The number of iterations used for each system is reported in Table 1. In this and all subsequent tables and figures, DFT denotes the ωB97X-D3/def2-TZVP level.

**Table 1:** Summary of Low-Level Exploration and High-Level Refinement Statistics for the Five Test Systems

	MEA	cBD–OH	cBD-CN	cBD-CCH	Tz2-HA
Low-level calculations					
iterations	30	50	50	50	3
tasks	60	60	60	10	10
reactions	696	1198	1079	437	186
number of reactions after high-level refinement stage					
DFT	435	796	712	266	100
r^2^SCAN-3c ML-CC	447	747	652	202	115
UMA	424	850	830	327	121
MACE-OMOL-0	405	821	775	288	128

Each iteration consists of high-temperature MD trajectory generation, followed by structure optimization, enabling the identification of minima and TS candidates on the low-level PES. All exploratory simulations were performed by using the PM7 semiempirical method.^30^ The structures obtained from each iteration are passed to the structure recognition and classification stage, where minima, TSs, reactants, and products are identified and assembled into the preliminary reaction connectivity maps. The reaction events collected over all iterations are aggregated to construct the complete low-level RXN for each system. The total number of elementary reactions identified at this stage is reported in Table 1. For all reaction-network explorations, we enabled the option to identify barrierless reactions leading to fragments containing two or more atoms. Consequently, for simplicity, barrierless H atom eliminations were excluded.

### 3.2 High-Level Refinement

The PM7-derived RXNs were refined using four high-level approaches: (i) DFT calculations at the ωB97X-D3/def2-TZVP level, (ii) the recently developed Δ-ML r^2^SCAN-3c ML-CC approach,^10^ (iii) the uma-m-1p1 model from the UMA family of MLIPs, and (iv) the MACE-OMOL-0 model. The latter three approaches were integrated into the AutoMeKin workflow as described in Section 2, enabling their seamless use in geometry optimization, transition-state refinement, and MEP characterization. The resulting number of reactions for each system and refinement approach is summarized in Table 1.

Geometry optimizations and MEP calculations with the ML potentials were performed using the Sella optimizer interfaced with ASE. Geometry optimizations (TSs and minima) employed a maximum force threshold of 0.005 eV Å^–1^, whereas MEP calculations used a step size of 0.08 Å u^1/2^ and a maximum force convergence criterion of 0.01 eV Å^–1^. Vibrational frequencies were computed numerically with the ASE Vibrations module.

For the DFT calculations, geometry optimizations and MEP calculations were performed by default using the native ORCA optimizers, with Hessians computed every ten steps during MEP evaluation. For a subset of reactions, however, the same calculations were also carried out with ASE/Sella to enable a more direct comparison to the ML-based reaction networks. In this case, the Sella parameters described above were employed without change.

As reference data for assessing the performance of approaches (i)–(iv), we carried out single-point CCSD­(T)-F12a/cc-pVDZ-F12//ωB97X-D3/def2-TZVP calculations for the MEA system. For open-shell species, restricted open-shell Hartree–Fock (ROHF) references were employed within the DLPNO–CCSD­(T)-F12a framework to minimize spin contamination.^31–33^ The DLPNO approximation was also used for the larger Tz2-HA system. All reference BHs and REs include zero-point vibrational energy (ZPE) corrections computed at the ωB97X-D3/def2-TZVP level.

## 4 Results and Discussion

The performance of the four high-level refinement approaches, DFT, r^2^SCAN-3c ML-CC, UMA, and MACE-OMOL-0, is assessed by comparing the energy profiles along the main reaction channels of each network to reference values. For MEA and Tz2-HA, the comparison is further extended to decomposition kinetics through RRKM calculations, which probe how differences in barrier heights and vibrational frequencies propagate into product population fractions. The results are discussed separately for each test system in the following sub-sections.

### 4.1 Monoethanolamine (MEA)

This molecule, also named 2-aminoethanol, is a small neutral CHON-containing molecule included in the OMol25 (3649 entries) and Green’s RDB7 datasets. The latter database contains only three entries, corresponding to MEA conformers, and does not include any of the reactions involved in the unimolecular decomposition of this system. Previous gas-phase computational studies of MEA have mainly focused on atmospheric oxidation, including OH-initiated H-abstraction, MEA-derived radical chemistry, O_2_ addition/elimination pathways, and nitrogen-containing products relevant to environmental emissions.^34–37^ Here, we investigate the unimolecular decomposition of MEA, a process of direct relevance to combustion chemistry; to our knowledge, this issue has not been specifically addressed in previous studies.

As shown in Table 1, the low-level exploration of the global reactive PES of MEA predicted approximately 700 elementary reactions. After high-level refinement, the total number of elementary steps decreased to 400–450, depending on the method employed. We note that the reactions discovered by AutoMeKin comprise fragmentations, isomerizations or conformational interconversions, and processes classified as bimolecular reactions. Some of the steps identified as bimolecular may correspond to transformations involving van der Waals complexes, such as fragment reorientations. In this study, the bimolecular processes were not analyzed.

We used two screening procedures to assess reaction matching among the approaches considered in this work. The first criterion is based on adjacency matrices. The second criterion builds on the first by also considering the root-mean-square deviation (RMSD) between the geometries being compared, thereby discriminating between reactions involving different conformers. Additional details of the screening criteria are provided in the Supporting Information, and Figure S1 shows the corresponding workflow. The reactions identified by UMA, MACE-OMOL-0, and r^2^SCAN-3c that match those in the DFT ωB97X-D3/def2-TZVP network are reported in Table S1 for all systems studied in this work.

Using the tighter criterion, denoted as S-2 in Table S1, the number of reaction matches with the DFT network for the MEA system is 130, 76, and 153 for UMA, MACE-OMOL-0, and r^2^SCAN-3c, respectively. Taking the CCSD­(T)-F12a/cc-pVDZ-F12//ωB97X-D3/def2-TZVP results as reference, the mean absolute errors (MAEs) of BHs and REs for these common reactions are shown in Figure S2 of the Supporting Information. For the DFT MAEs, all reactions in the DFT network were included. Except for the DFT MAE calculated for the barrier heights of the reverse reactions, all MAEs are below 2 kcal mol^–1^, confirming the good accuracy of these approaches.

For this system, we also compared the DFT networks generated using the saddle point optimization algorithm implemented in ORCA^8,9^ and the Sella optimizer,^29^ as described in the previous section. When the latter algorithm is used, the resulting network contains 421 reactions, that is, 14 fewer than the network obtained with the standard ORCA optimizer. Application of the S-1 and S-2 matching criteria to the two DFT networks indicates that 295 and 118 reactions, respectively, are common to both networks. Although these results, together with those discussed above, show that the predicted network is sensitive to both the optimization algorithm and the method used to describe the PES, all networks generally retain kinetically relevant pathways. Thus, the different methods predict essentially the same reaction mechanism.

Figure 2 depicts the five most important competing channels in the dissociation of MEA. The selection of these channels is based on the product abundances obtained from the automated kinetic study performed at the reference level, using an excitation energy of 150 kcal mol^–1^, with geometries and vibrational frequencies calculated at the ωB97X-D3/def2-TZVP level, and electronic energies determined from single-point CCSD­(T)-F12a/cc-pVDZ-F12 calculations. The figure includes the energies of the stationary points, relative to the most stable MEA conformer and corrected with the ZPE contributions. Two of these reactions lead to stable products, namely, channel 1 (acetaldehyde + ammonia) and channel 2 (PR1: ethenamine + water). In the remaining reactions, at least one of the products is a reactive species, either a radical or a carbene. We note that the reaction diagrams presented for all systems investigated in this work are schematic; pathways involving different conformers of the reactants, intermediates, and TSs, where present, are not explicitly shown.

**Figure 2:**
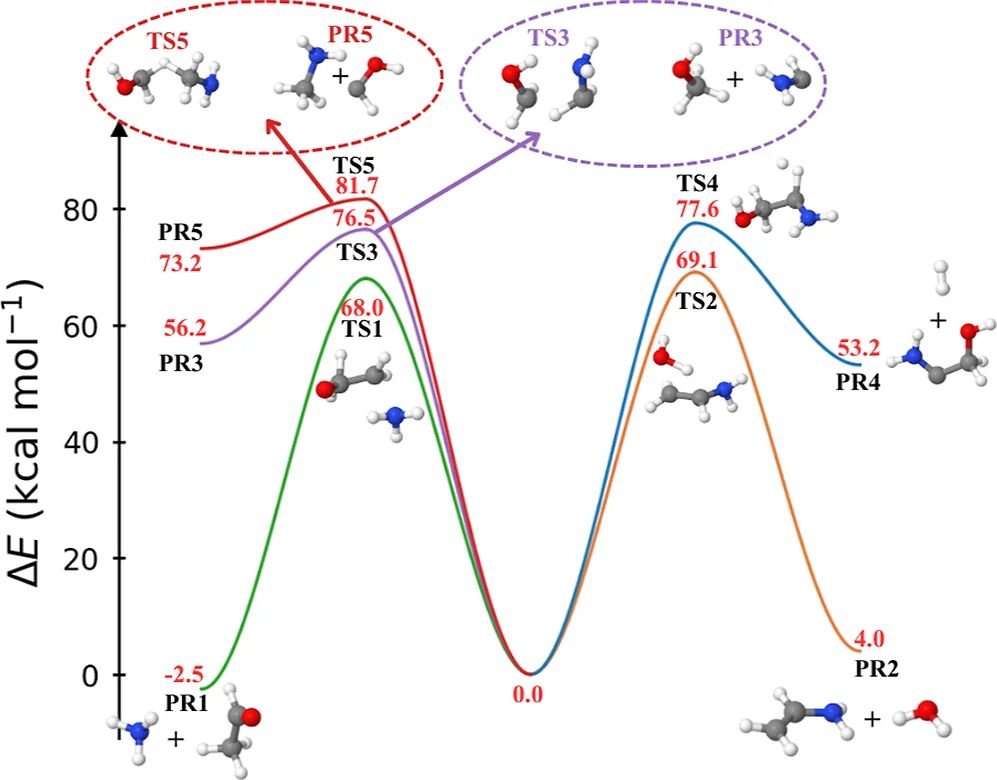
Energy profiles of the five most relevant fragmentation channels in the unimolecular decomposition of MEA. Reference energies (in kcal mol^–1^) are relative to the most stable conformer of MEA and include ZPE contributions. Ball-and-stick structures are shown for selected stationary points. For visual clarity, TS3 and TS5, together with their associated products, are enclosed in dashed ellipses.

The five channels discussed above were also predicted by all other approaches considered in this study. Table 2 summarizes the deviations of the relative energies obtained with the different high-level model chemistries from the corresponding reference values, namely, the CC single-point results. The values in this and the remaining tables are calculated as the difference between the high-level results and the corresponding reference values, with ZPE contributions included. Boldface values indicate the method yielding the smallest absolute deviation for each stationary point (TS or product). In general, the absolute deviations are below 2 kcal mol^–1^, further supporting the excellent accuracy of these approaches. The deviations obtained with UMA and MACE-OMOL-0 are very similar, as expected given that both ML models were trained on the OMol25 database.^18^

**Table 2:** Energy Deviations (in Kcal mol^–1^) with Respect to CCSD­(T)-F12a for the Stationary Points of the Relevant Channels of the MEA System^a^

configuration	DFT	UMA	MACE-OMOL-0	r^2^SCAN-3c ML-CC
TS1	–**0.23** (67.79)	–1.37 (66.65)	–1.81 (66.21)	2.06 (70.08)
TS2	0.64 (69.76)	–2.03 (67.09)	–2.37 (66.75)	**0.09** (69.21)
TS3	1.16 (77.63)	0.41 (76.88)	**0.00** (76.47)	1.30 (77.77)
TS4	–**0.32** (77.24)	–1.59 (75.97)	–1.67 (75.89)	0.68 (78.24)
TS5	1.76 (83.45)	0.66 (82.35)	0.67 (82.36)	–**0.36** (81.33)
PR1	0.87	1.03	0.98	**0.44**
PR2	3.40	1.39	1.39	**0.37**
PR3	0.95	–0.42	–0.38	**0.36**
PR4	1.07	–0.97	–1.04	**0.08**
PR5	1.64	**–0.13**	–0.21	0.28

a The ZPE-corrected energy of the most stable conformer of MEA was used as the reference zero. Values in parentheses correspond to the TS energies, i.e., the BHs. Bold values indicate the smallest absolute deviation for each configuration.

Barrier heights exert a critical influence on the kinetics of chemical reactions. However, other factors, such as the vibrational frequencies of reactants and transition states, may also play an important role, with their influence becoming more pronounced under high-temperature or high-energy conditions, where entropic contributions become increasingly significant. It is therefore worthwhile to examine this effect in the present system. Given the complexity of the global reaction network, from which clear mechanistic conclusions may be difficult to extract, we focus here on the five most relevant competing reactions, shown in Figure 2. To this end, we calculated energy-specific rate coefficients, *k*(*E*), using Rice–Ramsberger–Kassel–Marcus (RRKM) theory:^38^

1 $$k\left(E\right)=\sigma \frac{W^{‡}\left(E-E_{0}\right)}{h\rho \left(E\right)}$$

where σ is the reaction-path degeneracy (1 for all reactions studied here), *W* ^‡^(*E* – *E*_0_) is the sum of states of the transition state, *E*_0_ is the barrier height, and ρ­(*E*) is the density of states of the reactant.

The RRKM calculations were performed using the kinetics script of AutoMeKin, in which sums and densities of states are evaluated by direct counting with the Beyer–Swinehart algorithm.^39^ In the calculations reported here, the state count is constructed from the harmonic vibrational frequencies of the reactants and transition states and therefore assumes independent harmonic-oscillator energy levels. Accordingly, the kinetic results obtained with this implementation should be regarded as primarily qualitative or semiquantitative, especially when low-frequency vibrational modes contribute differently to the state counts of the reactants and transition states. Such modes may represent hindered internal rotations or other large-amplitude motions for which the harmonic approximation can yield inaccurate densities and sums of states.^40,41^ A more accurate state count for these modes may require an explicit treatment of vibrational and torsional anharmonicity. Depending on the physical character of each mode, this may involve replacing the harmonic-oscillator description with a free-rotor treatment or with a hindered-rotor model. The latter may be treated using analytical or semiclassical approximations or, at a higher level of rigor, by calculating the torsional potential and numerically solving the associated Schrödinger equation to obtain the torsional energy levels.^42,43^ When several torsions or low-frequency motions are strongly coupled, multidimensional or multistructural approaches may be required instead of independent one-dimensional rotor corrections.^44,45^ Finally, more rigorous quantitative rate calculations may also require a variational treatment of the transition state to account for recrossing and the inclusion of quantum-mechanical tunneling, particularly for reactions involving the transfer of light atoms.^46,47^

Figure 3 displays the product population fractions as a function of the excitation energy, *E*, for the five competing unimolecular channels shown in Figure 2. For each channel, the population fraction is computed as *k*_*i*_(*E*)/∑_*i*_ *k*_*i*_(*E*), where *k*_*i*_(*E*) is the rate coefficient of channel *i* and the denominator corresponds to the sum of the five individual rate coefficients. In each panel, the dashed lines correspond to the product populations obtained at the reference CCSD­(T)-F12a/cc-pVDZ-F12//ωB97X-D3/def2-TZVP level of theory. At low excitation energies, the product population distribution for MEA decomposition is primarily governed by the BHs. Accordingly, the channel leading to acetaldehyde and ammonia is predicted to be dominant, consistent with its lowest barrier height (reference BH = 68.0 kcal mol^–1^), followed by the channel yielding ethenamine and water (reference BH = 69.1 kcal mol^–1^). Thus, in this low-energy regime, the product population fractions follow the order expected from the corresponding BHs. As the excitation energy increases, entropic effects become increasingly important, leading to a crossing of the two dominant product populations at 106 kcal mol^–1^, above which the ethenamine + water channel becomes favored. This behavior arises from the different contributions of the vibrational modes to the TS sum of states, *W* ^‡^(*E* – *E*_0_), particularly those associated with low- and mid-frequency modes. As shown in Table S2 of the SI, TS2 has a larger number of vibrational modes with frequencies substantially lower than the corresponding modes of TS1 than vice versa; this is the case, for example, for modes ν_8_–ν_11_. Consequently, the sum of states of TS2 grows more rapidly with excitation energy than that of TS1. The product populations associated with the remaining fragmentation channels also increase with excitation energy, although they remain minor throughout the energy range investigated.

**Figure 3:**
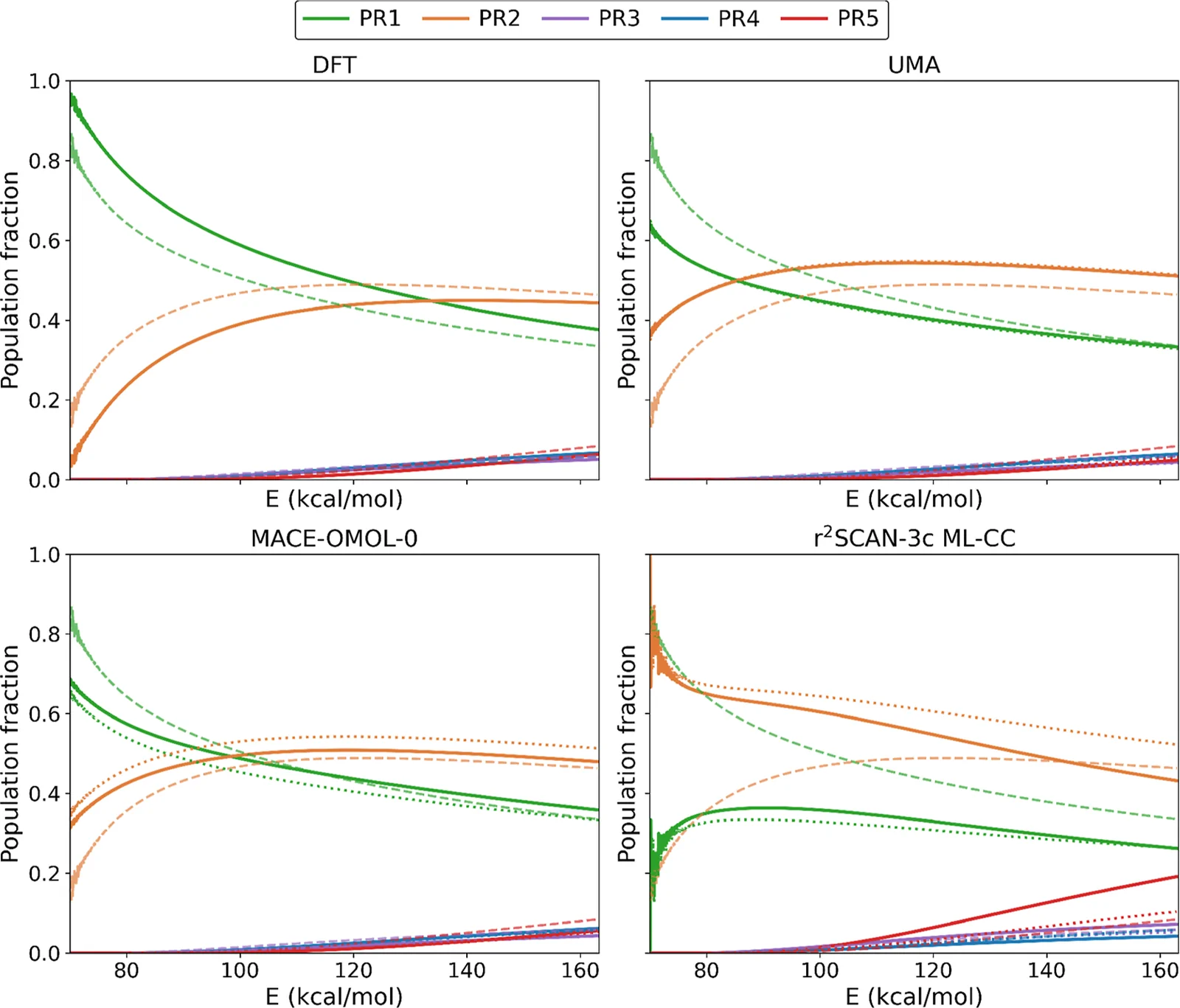
Product population fractions for MEA decomposition as a function of excitation energy for the competing channels depicted in Figure 2. The dashed lines in each panel correspond to the reference-level populations obtained from CCSD­(T)-F12a/cc-pVDZ-F12//ωB97X-D3/def2-TZVP calculations. For UMA, MACE-OMOL-0, and *r* ^2^SCAN-3c ML-CC, the dotted lines correspond to RRKM calculations performed using the ωB97X-D3/def2-TZVP frequencies.

The populations calculated at the DFT ωB97X-D3/def2-TZVP level somewhat overestimate the formation of acetaldehyde and ammonia. At this level of theory, the population crossing occurs at 134 kcal mol^–1^. This overestimation of the channel 1 population can be readily rationalized in terms of the energy deviations reported in Table 2 for the relevant stationary points. Specifically, the DFT level slightly underestimates the BH of channel 1 while overestimating that of channel 2. Both UMA and MACE-OMOL-0 underestimate the BHs of channels 1 and 2, with the underestimation being more pronounced for channel 2 in both cases. This leads to barrier heights that are more similar to each other. As a result, the population crossing points predicted with these MLIPs are shifted to lower excitation energies, namely, 86 and 98 kcal mol^–1^ for UMA and MACE-OMOL-0, respectively. Among the tested approaches, the best agreement with the reference populations is obtained with MACE-OMOL-0. In contrast, the populations calculated with the r^2^SCAN-3c ML-CC method show the poorest agreement with the reference results. In fact, at this level of theory, channel 2 dominates over the entire range of excitation energies considered in this analysis. This discrepancy arises from the overestimation of the channel 1 barrier by approximately 2 kcal mol^–1^, which places this barrier 0.9 kcal mol^–1^ above that of channel 2.

Finally, to assess the influence of the vibrational frequencies on the calculated rate coefficients and, consequently, on the product abundances, the RRKM rate coefficients for the MLIPs and the *r* ^2^SCAN-3c ML-CC approach were recalculated using the DFT vibrational frequencies. The resulting populations are shown in Figure 3 as dotted lines. For UMA, the results remain essentially unchanged, indicating a close agreement between the ωB97X-D3/def2-TZVP and UMA vibrational frequencies for this system. For MACE-OMOL-0, replacing the original vibrational frequencies with the DFT values slightly increases the population of the channel yielding ethenamine and water, with a concomitant decrease in the population of the channel leading to acetaldehyde and ammonia. A similar behavior is found for the r^2^SCAN-3c ML-CC method, for which a noticeable decrease in the population of the channel 5 products is also observed at high excitation energies.

### 4.2 Cyclobutadiene-Derived Radicals (cBD–OH, cBD-CN, and cBD-CCH)

Largo et al.^48^ investigated the gas-phase reactions

$$c‐C_{4}H_{4}+X\to c‐C_{4}H_{3}X+H,\left(X=OH,\;CN,\;\text{and}\;CCH\right)$$

at the CCSD­(T)-F12/cc-pVTZ-F12//B2PLYPD3/aug-cc-pVTZ level to analyze whether the hydroxyl (OH), cyano (CN), and ethynyl (CCH) derivatives of the highly reactive, antiaromatic cyclobutadiene (c-C_4_H_4_) can readily form in the interstellar medium, provided that c-C_4_H_4_ is present there. For OH addition, they identified a barrierless step leading to two deeply bound intermediates (denoted as I1 and I2 in their work), from which syn- and anti-*c*-C_4_H_3_OH are formed through pathways exhibiting submerged barriers. As shown in Table 1, the low-level exploration yielded a total of 1198 elementary steps. After high-level refinement, this number decreased significantly, with the final number of reactions ranging from 747 for r^2^SCAN-3c ML-CC to 850 for UMA. The four high-level reactive PESs exhibit the same entrance channel reported by Largo et al.^48^ and feature the same key intermediates (denoted as cBD–OH and MIN3 in our nomenclature, corresponding to I1 and I2 in ref 48). As shown in Figure 4, at the reference level, the most stable conformers of cBD–OH and MIN3 lie 72.3 and 75.0 kcal mol^–1^ below the reactant asymptote, respectively, and are formed without passing through any barrier. Furthermore, our simulations suggest barrierless formation of *syn*- and *anti*-*c*-C_4_H_3_OH from c-C_4_H_4_ + OH. Although the identification of barrierless channels in AutoMeKin relies on the topology of the underlying semiempirical potential energy surface and should therefore be interpreted with some caution, the agreement with the high-level study of Largo et al.^48^ provides strong support for the reliability of the predicted mechanisms.

**Figure 4:**
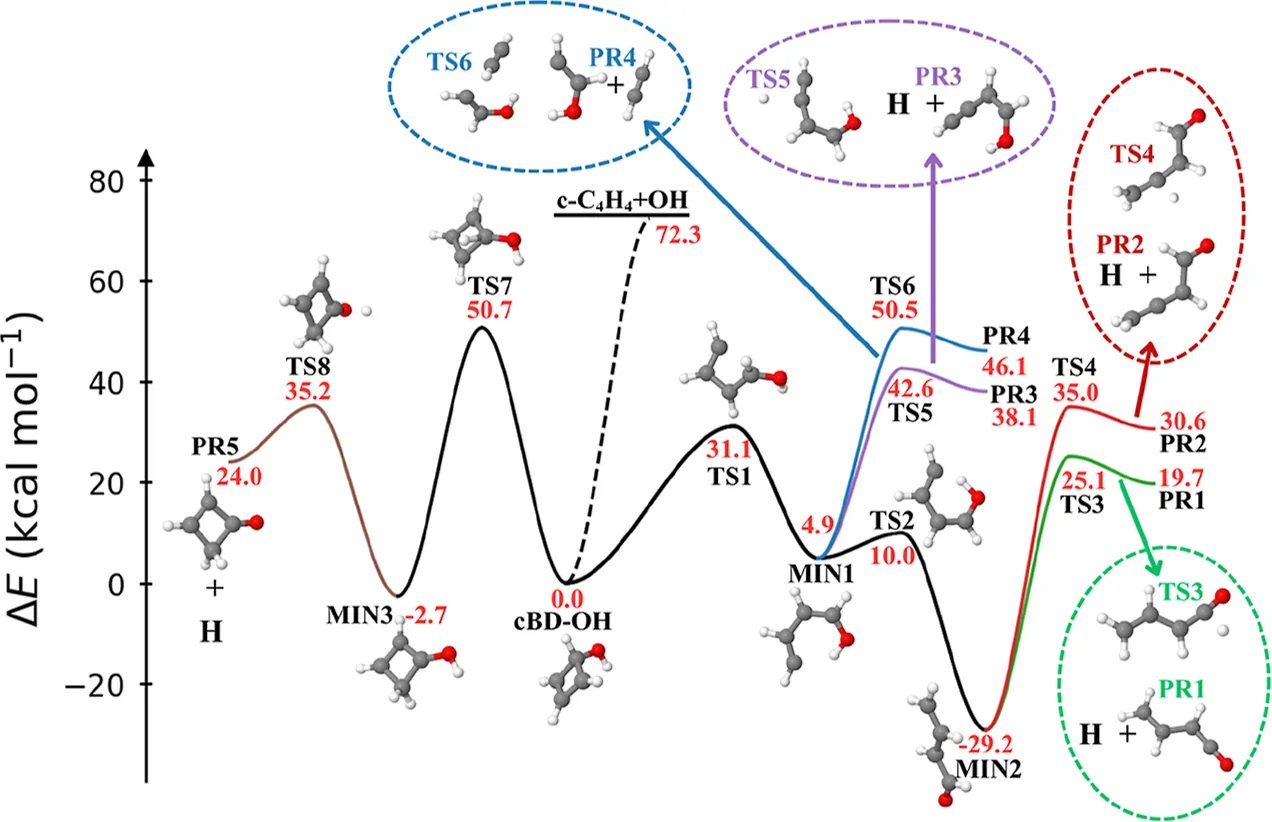
Simplified potential energy diagram for the c-C_4_H_4_ + OH reaction system obtained from the AutoMeKin exploration. Energies (in kcal mol^–1^) are ZPE-corrected DLPNO–CCSD­(T)-F12a reference values relative to the most stable conformer of cBD–OH.

Beyond the two pathways previously reported by Largo et al.,^48^ the automated exploration reveals additional reactions connected to the initial adducts through submerged transition states. The profiles for the most relevant channels are depicted in Figure 4. As shown in the figure, cBD–OH can undergo H-atom migration to form an allylic cyclobutenol radical (MIN3, −2.7 kcal mol^–1^ relative to cBD–OH), from which elimination of the hydroxyl hydrogen atom yields cyclobutenone (PR5, 24.0 kcal mol^–1^). Alternatively, opening of the highly strained four-membered ring of cBD–OH gives the 4-hydroxybuta-1,3-dien-1-yl radical (MIN1, 4.9 kcal mol^–1^), which can subsequently isomerize to the 4-formylbut-2-en-1-yl radical (MIN2, −29.2 kcal mol^–1^). Two competing fragmentation processes are available from MIN1: one leading to H + (*Z*)-but-1-en-3-yn-1-ol (PR3, 38.1 kcal mol^–1^) and the other yielding acetylene + ethen-1-ol-2-yl radical (PR4, 46.1 kcal mol^–1^). Finally, H-elimination from MIN2 leads to either vinylketene (PR1, 19.7 kcal mol^–1^) or buta-2,3-dienal (PR2, 30.6 kcal mol^–1^). Because all TSs along these pathways lie well below the reactant asymptote, all five channels are energetically accessible under interstellar conditions and may contribute to the chemistry of the OH + cyclobutadiene system.

For this system, the overall performance of the four potential energy models, assessed against the DLPNO–CCSD­(T)-F12a/cc-pVDZ-F12//ωB97X-D3/def2-TZVP reference values, is shown in Figure S3 of the Supporting Information. In this figure, the MAEs were calculated for the reactions that fulfill the S-2 screening criterion, as specified in Table S1 of the Supporting Information. In general, the MAEs are smaller than 2 kcal mol^–1^, indicating good agreement with the reference method. A more detailed comparison with the reference results is provided in Table 3 for the stationary points depicted in Figure 4. Compared with the results obtained for MEA, significant discrepancies are found for several stationary points. For example, the ωB97X-D3/def2-TZVP calculations show relatively large errors, of about 5 kcal mol^–1^, for c-C_4_H_4_ + OH, TS5, TS6, and PR4. UMA exhibits no absolute deviations larger than 4 kcal mol^–1^. MACE-OMOL-0 shows sizeable absolute deviations for TS1, TS5, and TS7, comparable to those obtained with DFT. The r^2^SCAN-3c ML-CC approach performs well overall, although deviations of up to 4 kcal mol^–1^ are observed for the product energies; notably, however, this method yields the smallest deviation for PR4 among the four approaches. In any case, our aim is not to provide a rigorous comparison of the accuracy of the four high-level approaches employed in this work; rather, it is to show that UMA and MACE-OMOL-0 can serve as surrogates for DFT methods.

**Table 3:** Deviations from CC Reference Energies (kcal mol^–1^) for Stationary Points along the Main Channels of the cBD–OH System^a^

configuration	DFT	UMA	MACE-OMOL-0	r^2^SCAN-3c ML-CC
c-C_4_H_4_ + OH	5.81	3.36	3.87	**2.03**
MIN1	1.03	**0.70**	1.14	–1.16
MIN2	–0.96	–0.09	**–0.08**	1.50
MIN3	–2.25	–1.19	–0.76	**–0.10**
TS1	2.18	1.79	4.35	**1.39**
TS2	**–0.81**	–1.11	–2.61	–1.55
TS3	0.56	–0.43	–1.07	**0.25**
TS4	2.02	**0.45**	1.08	0.87
TS5	5.57	2.19	5.94	**1.25**
TS6	4.05	1.58	1.69	**–0.11**
TS7	–2.11	–2.46	–4.72	**1.14**
TS8	**–0.25**	–1.79	–0.94	–0.93
PR1	–0.96	**–0.02**	–1.20	4.08
PR2	**–0.23**	1.48	0.26	3.73
PR3	2.89	**0.44**	–0.76	3.70
PR4	5.42	3.81	3.55	**0.39**
PR5	–1.26	**–1.02**	–2.15	3.61

a The ZPE-corrected energy of the most stable cBD–OH conformer was used as the reference zero. Bold values indicate the smallest absolute deviation for each configuration.

As described in the work of Largo et al.,^48^ the association reaction between cyclobutadiene and the cyano radical proceeds without an entrance barrier, leading to the cBD-CN radical (I1 in ref 48), as shown in Figure 5. This radical can isomerize to an allylic cyclobutenecarbonitrile radical (MIN1, corresponding to I2 in ref 48), which is 5.9 kcal mol^–1^ more stable than the cBD-CN radical, including ZPE corrections, at the reference level of theory employed in the present study. The calculations reported by Largo et al.^48^ indicate that both minima can undergo H atom elimination to form c-C_4_H_3_CN through loose TSs. In the present work, however, H atom elimination from cBD-CN (PR7) was obtained only in the high-level calculations using the UMA potential, as indicated by the blue-stone dashed line. Notably, the low-level exploration carried out with the PM7 semiempirical method did not predict this channel. This apparent discrepancy can be rationalized by the fact that high-level TS optimizations initiated from low-level TS structures may converge to a different nearby saddle point on the PES.

**Figure 5:**
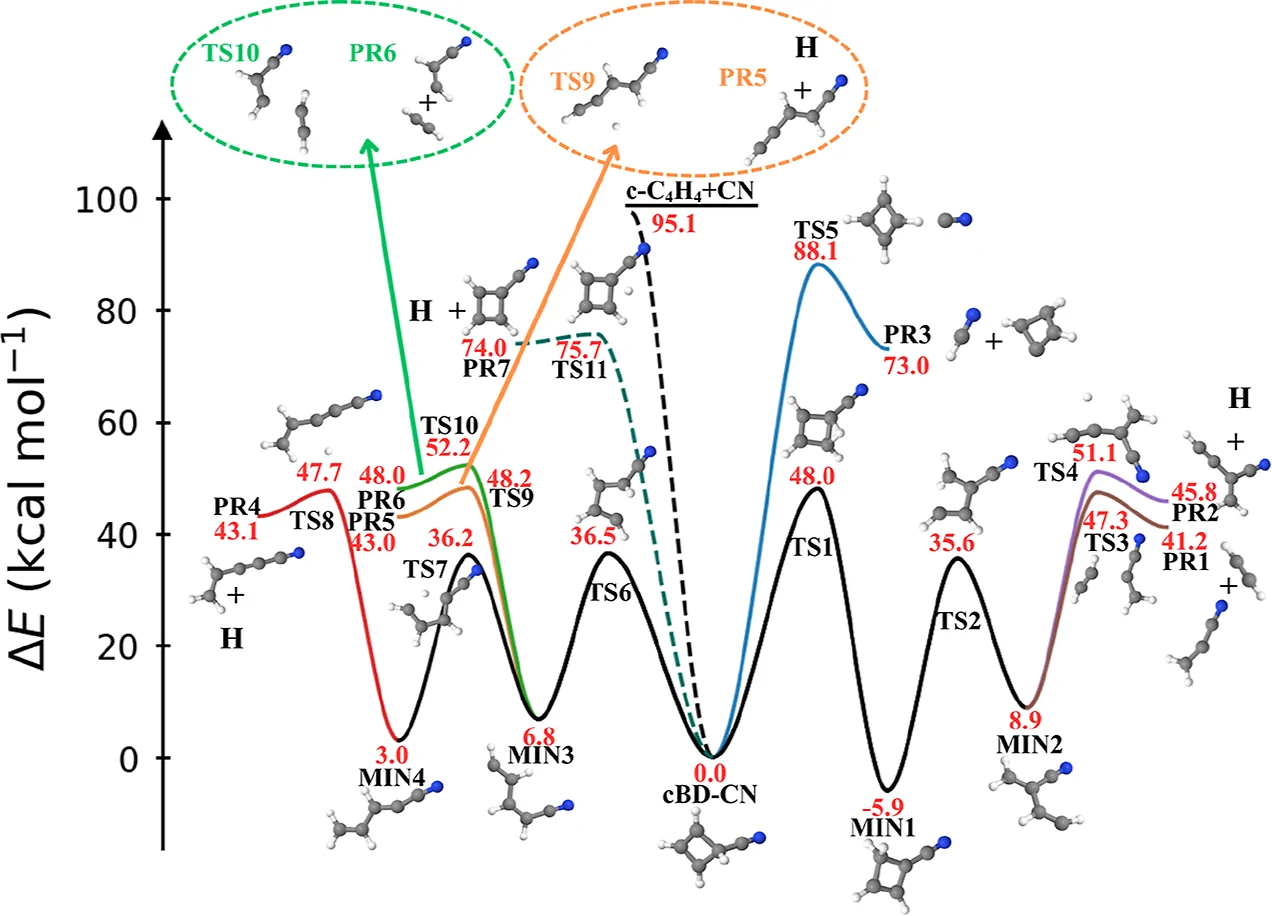
Simplified potential energy diagram for the c-C_4_H_4_ + CN reaction system obtained from the AutoMeKin exploration. Energies (in kcal mol^–1^) are ZPE-corrected DLPNO–CCSD­(T)-F12a reference values relative to cBD-CN.

The remaining channels depicted in Figure 5 were obtained with the four high-level approaches used in this study. The AutoMeKin exploration predicts direct elimination of hydrogen cyanide, yielding a highly reactive four-membered ring as the accompanying fragment. Although the TS associated with this fragmentation (TS5) has a relatively high energy barrier (88.1 kcal mol^–1^), this channel might be relevant under high-energy conditions. The other fragmentations shown in the figure involve a series of consecutive steps. In the right-hand branch of the diagram, an H atom migration in cBD-CN leads to MIN1 (−5.9 kcal mol^–1^), the analogue of MIN3 in cBD–OH. This minimum may then undergo ring opening, resulting in the 2-cyanobuta-1,3-dien-4-yl radical (MIN2, 8.9 kcal mol^–1^). From MIN2, two competing reactions can occur: formation of acetylene and the cyanoethenyl radical (PR1, 41.2 kcal mol^–1^) and H atom elimination to form 2-methylidenebut-3-ynenitrile (PR2, 45.8 kcal mol^–1^).

The channels displayed to the left of cBD-CN originate from ring opening of cBD-CN, yielding the 1-cyanobuta-1,3-dien-4-yl radical (MIN3, 6.8 kcal mol^–1^). This minimum may undergo H atom elimination to form (*E*)-pent-2-en-4-ynenitrile (PR5, 43.0 kcal mol^–1^), or it may fragment to yield acetylene and the 2-cyanoethen-1-yl radical (PR6, 48.0 kcal mol^–1^). Alternatively, MIN3 may isomerize to the 1-cyanobuta-1,3-dien-1-yl radical (MIN4, 3.0 kcal mol^–1^). From this minimum, C–H bond dissociation can lead to pent-4-en-2-ynenitrile (PR4, 43.1 kcal mol^–1^). Note that all stationary points of the channels shown in Figure 5 have energies well below that of the c-C_4_H_4_ + CN entrance system, except for the direct fragmentations leading to PR3 and PR7. As already mentioned, the latter channel was obtained only with UMA.

Table 4 lists the energy deviations, relative to the ZPE-corrected DLPNO–CCSD­(T)-F12a reference values, obtained with the four high-level methods for the stationary points included in Figure 5. The ZPE-corrected energy of the cBD-CN structure was used as the reference zero. A first noteworthy result is the large deviation (≈10 kcal mol^–1^) found for the c-C_4_H_4_ + CN entrance system with both the DFT level employed in this work, namely, ωB97X-D3/def2-TZVP and UMA. As mentioned in the Introduction section, except for the MEA system, the remaining systems considered here are not included in the OMol25 dataset. Therefore, significant deviations from the DLPNO–CCSD­(T)-F12a reference values may be expected, particularly for reactive configurations that are poorly represented in the corresponding training data.

**Table 4:** Deviations from CC Reference Energies (kcal mol^–1^) for Stationary Points along the Main Channels of the cBD-CN System^a^

configuration	DFT	UMA	MACE-OMOL-0	r^2^SCAN-3c ML-CC
c-C_4_H_4_ + CN	9.94	10.88	3.15	**2.16**
MIN1	–1.44	–0.97	**–0.40**	0.97
MIN2	3.64	2.41	2.00	**0.76**
MIN3	2.96	1.97	1.80	**1.27**
MIN4	1.12	1.05	**0.47**	**0.47**
TS1	–1.58	–2.59	–2.30	**0.75**
TS2	2.97	2.40	4.11	**0.65**
TS3	4.70	2.12	**1.96**	2.05
TS4	7.49	3.65	2.77	**1.74**
TS5	0.89	**0.64**	2.29	–2.57
TS6	3.16	2.60	5.10	**1.05**
TS7	2.66	1.49	**1.41**	2.61
TS8	6.24	3.65	3.58	**0.98**
TS9	6.64	3.05	3.25	**1.10**
TS10	4.97	2.28	1.37	**0.90**
TS11	-	1.85	-	-
PR1	4.33	3.22	3.19	**0.75**
PR2	5.14	2.13	**0.62**	4.40
PR3	2.92	2.81	1.68	**–0.49**
PR4	3.93	1.85	**0.41**	4.23
PR5	4.31	1.67	**0.17**	3.96
PR6	6.21	4.61	4.00	**0.52**
PR7	-	1.94	-	-

a The ZPE-corrected energy of the cBD-CN structure was used as the reference zero. Bold values indicate the smallest absolute deviation for each configuration.

The results obtained with ωB97X-D3/def2-TZVP also exhibit large deviations, exceeding 4 kcal mol^–1^, for many of the stationary points. Overall, the performance of the UMA model is good, with only five stationary points showing absolute deviations of about 3–4 kcal mol^–1^, apart from the large deviation found for c-C_4_H_4_ + CN. For MACE-OMOL-0, seven stationary points exhibit absolute deviations larger than 3.0 kcal mol^–1^. The largest deviation obtained with this foundation model, 5.1 kcal mol^–1^, occurs for TS6. The r^2^SCAN-3c ML-CC method performs very well overall, with only three stationary points, namely, PR2, PR4, and PR5, showing deviations of ≈4 kcal mol^–1^. This level of agreement is not unexpected, since the reference method employed in this work corresponds to that of the RDB7 dataset,^16,17^ which was used to construct the training set of our Δ-ML model. Nevertheless, the present systems are not represented in the RDB7 data. Most deviations shown in Table 4 are positive, indicating that the high-level methods tend to overestimate the reference-level relative energies when cBD-CN is used as the reference zero. Finally, as shown in Figure S4 of the SI, the MAEs calculated for all reactions filtered using the S-2 screening criterion (see Table S1) indicate somewhat larger deviations than those obtained for cBD–OH.

For the c-C_4_H_4_ + CCH system, we performed a less exhaustive AutoMeKin exploration than for the hydroxyl and cyano derivatives, using 10 tasks instead of 60 (see Table 1). Overall, the low-level exploration yielded 437 elementary reactions, which decreased after high-level refinement to 202 reactions for r^2^SCAN-3c ML-CC and 327 for UMA, corresponding to the smallest and largest final networks, respectively. The overall performance of the high-level approaches employed in this work is shown in Figure S5 of the Supporting Information. As for the other systems, only the reactions that passed the S-2 screening criterion were considered; the corresponding numbers are reported in Table S1 of the Supporting Information. As shown in Figure S5, the barrier heights calculated with MACE-OMOL-0 exhibit MAEs close to 3 kcal mol^–1^. Overall, the performance of these approaches is reasonably good. For the ML-based methods, this agreement is particularly encouraging, considering that such systems are underrepresented in the training sets used to fit the corresponding models.

According to our AutoMeKin exploration, the most relevant channels that may arise from the barrierless association of cyclobutadiene with the ethynyl radical are shown in Figure 6. As observed for the cyano derivative, the UMA calculations predict direct cleavage of the C–H bond at the ethynyl-substituted ring carbon of the 1-ethynylcyclobut-3-en-2-yl radical, leading to the ethynylcyclobutadiene product (PR4, 71.2 kcal mol^–1^). This channel is depicted in the figure by a blue-stone dashed pathway. As in the previous system, it is worth noting that this channel was not predicted in the low-level exploration. Apart from this direct fragmentation, all high-level methods predict migration of the hydrogen atom attached to the ethynyl-substituted ring carbon to one of the adjacent C atoms, forming MIN1 (−3.8 kcal mol^–1^), as well as ring opening of cBD-CCH to yield the hexa-1,3-dien-5-yn-1-yl radical (MIN4, 5.4 kcal mol^–1^). H-atom elimination from MIN4 leads to (*E*)-hexa-3-en-1,5-diyne (PR5, 40.8 kcal mol^–1^). Alternatively, MIN4 may fragment to yield acetylene and the but-1-en-3-yn-1-yl radical (PR6, 46.0 kcal mol^–1^). At the r^2^SCAN-3c ML-CC level, this latter channel is predicted as barrierless.

**Figure 6:**
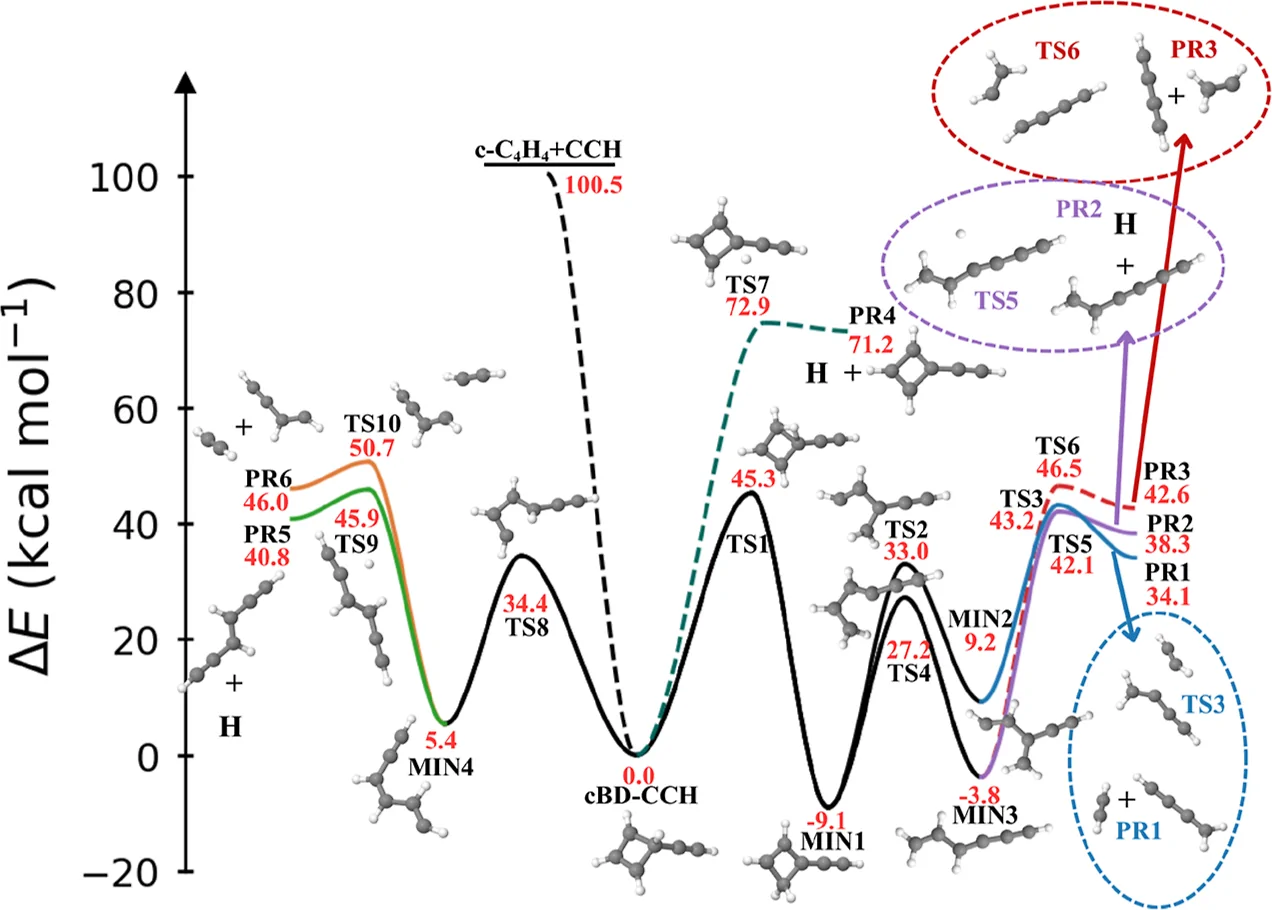
Simplified potential energy diagram for the c-C_4_H_4_ + CCH reaction system obtained from the AutoMeKin exploration. Energies (in kcal mol^–1^) are ZPE-corrected DLPNO–CCSD­(T)-F12a reference values relative to cBD-CCH.

MIN1 may undergo two competing ring-opening processes, leading to C_6_H_5_ radicals bearing terminal ethynyl groups, MIN2 and MIN3, with ZPE-corrected energies of 9.2 and −3.8 kcal mol^–1^ relative to cBD-CCH, respectively. MIN2 may further fragment to yield acetylene and a C_4_H_3_ radical with a terminal ethynyl group (PR1, 34.1 kcal mol^–1^). In contrast, MIN3 may undergo H-atom elimination to form hexa-1-en-3,5-diyne (PR2, 38.3 kcal mol^–1^). These fragmentation pathways were not located in the r^2^SCAN-3c calculations. Starting from MIN3, the ωB97X-D3/def2-TZVP calculations also predict fragmentation into diacetylene and the vinyl radical (PR3, 42.6 kcal mol^–1^), which is represented in the figure by a dashed red profile. This channel was not found with the other high-level methods, although the same products can be formed through alternative pathways, which generally involve high-energy profiles.

The transition state and the profile predicted with UMA for MIN3 → PR3 deserve special attention. The IRC calculated with this universal MLIP is shown in Figure S6 of the Supporting Information. Note that the TS geometry differs somewhat from that optimized at the DFT level (TS6, see Figure 6). For the IRC geometries, we performed single-point calculations at the DFT ωB97M-V/def2-TZVPD level^49–51^ employed in the construction of the OMol25 dataset.^18^ In the region with relative energies above 70 kcal mol^–1^, the DFT calculations show abrupt energetic deviations from the smooth UMA profile, together with SCF convergence problems. The DFT calculation for the black-circle point in Figure S6 that lies essentially on the UMA IRC gives an expectation value of ⟨*S* ^2^⟩ = 0.786, close to the ideal doublet value of 0.75. By contrast, the DFT calculation for the black-circle point that deviates strongly from the UMA profile gives a severely spin-contaminated solution, with ⟨*S* ^2^⟩ = 6.18, together with a very small HOMO–LUMO gap. In addition, a single-point DLPNO–CCSD­(T)-F12a calculation on the UMA transition-state geometry gave a T1 diagnostic of 0.048, and attempts to optimize the transition state at the ωB97M-V/def2-TZVPD level were unsuccessful. These observations suggest possible limitations of a single-reference description.

Complete active space self-consistent field (CASSCF) calculations^52^ were performed with ORCA^8,9^ to determine whether the TS optimized with UMA is an artifact or whether UMA describes this region of configuration space better than DFT. We employed the cc-pVTZ basis set^53^ and an active space of 11 electrons in 11 orbitals, comprising bonding and antibonding π molecular orbitals and the σ and σ* orbitals associated with the breaking C–C bond. Starting from the UMA-optimized geometry, optimization at the CASSCF­(11,11)/cc-pVTZ level yielded a similar TS structure. At this geometry, state-averaged CASSCF calculations including the two lowest-energy doublet, quartet, and sextet states (six in all) showed that the lowest-energy doublet had marked multiconfigurational character, with four configurations having weights greater than 0.10 and a maximum weight of 0.328. In contrast, the lowest-energy quartet and sextet states were each dominated by a single configuration, with weights of 0.846 and 0.821, respectively. The doublet–quartet energy gap was 0.647 eV. These results provide further evidence of the limitations of a single-reference description of the doublet state in this region of the potential energy surface and help explain the convergence difficulties and substantial spin contamination observed in the DFT calculations.

An intrinsic reaction coordinate (IRC) calculation performed at the CASSCF­(11,11)/cc-pVTZ level connected the TS to the same products predicted by UMA and to a reactant resembling the shoulder of the UMA IRC (Figure S7), which may correspond to an intermediate formed by a preceding H-atom migration. Thus, whereas UMA describes the reaction from the *Z* isomer of MIN3 as a single concerted but asynchronous elementary step involving H-atom migration followed by C–C bond cleavage, with a classical barrier of 88.9 kcal mol^–1^, the CASSCF results suggest a stepwise mechanism involving these two consecutive processes. The *Z* isomer of MIN3 was optimized at the CASSCF­(11,11)/cc-pVTZ level, and the electronic energies were refined using strongly contracted *N*-electron valence state second-order perturbation theory (SC-NEVPT2)^54–56^ and the cc-pVTZ basis set. For the ground-state doublet, the SC-NEVPT2 energy difference between the TS and the *Z* isomer of MIN3 was 79.5 kcal mol^–1^, comparable to the UMA barrier. The intermediate was 2.2 kcal mol^–1^ below the TS at the SA-CASSCF­(11,11)/cc-pVTZ level but 1.2 kcal mol^–1^ above it after the SC-NEVPT2 correction, indicating a relatively flat region of the potential energy surface. The optimized CASSCF TS is an early transition state along the C–C bond-cleavage coordinate, whereas the TS for the preceding H-atom migration is expected to be a late transition state along the corresponding reaction coordinate, assuming behavior similar to that reported for ethylene.^57^ Despite some differences between the UMA and multiconfigurational profiles, these results highlight a potential practical advantage of MLIPs, as accurate multireference data for regions where single-reference methods provide an inadequate description could be appropriately incorporated into training sets.

The differences between the high-level relative energies and the corresponding single-point DLPNO–CCSD­(T)-F12a/cc-pVDZ-F12 values are collected in Table 5. As can be seen, the results resemble those obtained for the cyano derivative. The largest deviations are found for the ωB97X-D3/def2-TZVP and UMA calculations on the c-C_4_H_4_ + CCH entrance system, namely, 7.9 and 6.0 kcal mol^–1^, respectively. In general, the DFT method shows the largest deviations. UMA and MACE-OMOL-0 outperform the ωB97X-D3/def2-TZVP level. The deviations obtained with r^2^SCAN-3c ML-CC are generally small, except for PR5. A drawback of this approach is that the baseline method failed to predict several channels that are relevant at the reference level. As observed for the cyano derivative, the high-level methods generally overestimate the reference-level relative energies, using cBD-CCH as the reference zero.

**Table 5:** Deviations from CC Reference Energies (kcal mol^–1^) for Stationary Points along the Five Main Channels of the cBD-CCH System^a^

configuration	DFT	UMA	MACE-OMOL-0	r^2^SCAN-3c ML-CC
c-C_4_H_4_ + CCH	7.89	6.02	**–0.80**	1.58
MIN1	–1.42	–0.83	**–0.65**	0.68
MIN2	3.42	2.01	1.40	**0.18**
MIN3	**0.72**	1.06	0.80	**0.72**
MIN4	3.04	2.02	1.76	**1.49**
TS1	–1.46	–2.14	–2.80	**0.18**
TS2	3.09	2.40	3.84	**0.68**
TS3	5.01	2.33	**1.65**	-
TS4	2.46	**1.73**	4.05	2.21
TS5	6.07	**3.21**	3.26	-
TS6	4.42	-	-	-
TS7	-	1.75	-	-
TS8	3.21	2.53	5.19	**0.82**
TS9	6.36	2.64	2.60	**0.95**
TS10	5.11	2.82	**1.67**	-
PR1	3.65	**2.64**	2.81	-
PR2	3.91	1.80	**0.15**	-
PR3	5.30	3.62	2.22	**0.93**
PR4	-	2.05	-	-
PR5	4.15	1.30	**–0.40**	3.72
PR6	6.19	4.47	3.84	**1.99**

a The ZPE-corrected energy of the CBC-CCH structure was used as the reference zero. Bold values indicate the smallest absolute deviation for each configuration.

### 4.3 *N*-[Bis­(2H-Triazol-4-yl)­methylidene]­hydroxylamine (C_5_H_5_N_7_O)

This compound is a nitrogen-rich bis-triazolyl oxime (Tz2-HA) that is not included in the OMol25 dataset, and its fragmentation PES involves molecular configurations that are likely poorly represented, if not entirely absent, in that dataset.^18^ Because of the size of this system, we did not carry out an exhaustive AutoMeKin exploration. As shown in Table 1, the low-level search identified 186 elementary steps at the semiempirical PM7 level. After high-level refinement, these were reduced to 100 steps at the DFT level and 128 steps at the MACE-OMOL-0 level, corresponding to the smallest and largest refined reaction networks, respectively.

Figure 7 displays the most relevant decomposition pathways according to the reference-level calculations. As for the previous systems, we present a simplified diagram in which only the most stable conformers are considered. In channel 1, cleavage of one of the triazolyl rings produces hydrogen cyanide as a co-product (PR1, 58.6 kcal mol^–1^). Channel 2 also involves triazolyl-ring cleavage, but in this case, it yields HCNNH as the co-product (PR2, 60.6 kcal mol^–1^). The remaining two channels proceed through fragmentation pathways that generate separated ring-containing products. In PR3 (85.8 kcal mol^–1^), one of the resulting rings corresponds to a highly reactive species. By contrast, channel 4 leads to two stable triazole derivatives (PR4, −18.6 kcal mol^–1^). Note that the BH for channel 3 (83.2 kcal mol^–1^) is 2.7 kcal mol^–1^ lower than the associated reaction energy (85.8 kcal mol^–1^). This apparent discrepancy arises because, at the DLPNO–CCSD­(T)-F12a/cc-pVDZ-F12//ωB97X-D3/def2-TZVP level, TS3 lies 3.2 kcal mol^–1^ below PR3 in electronic energy.

**Figure 7:**
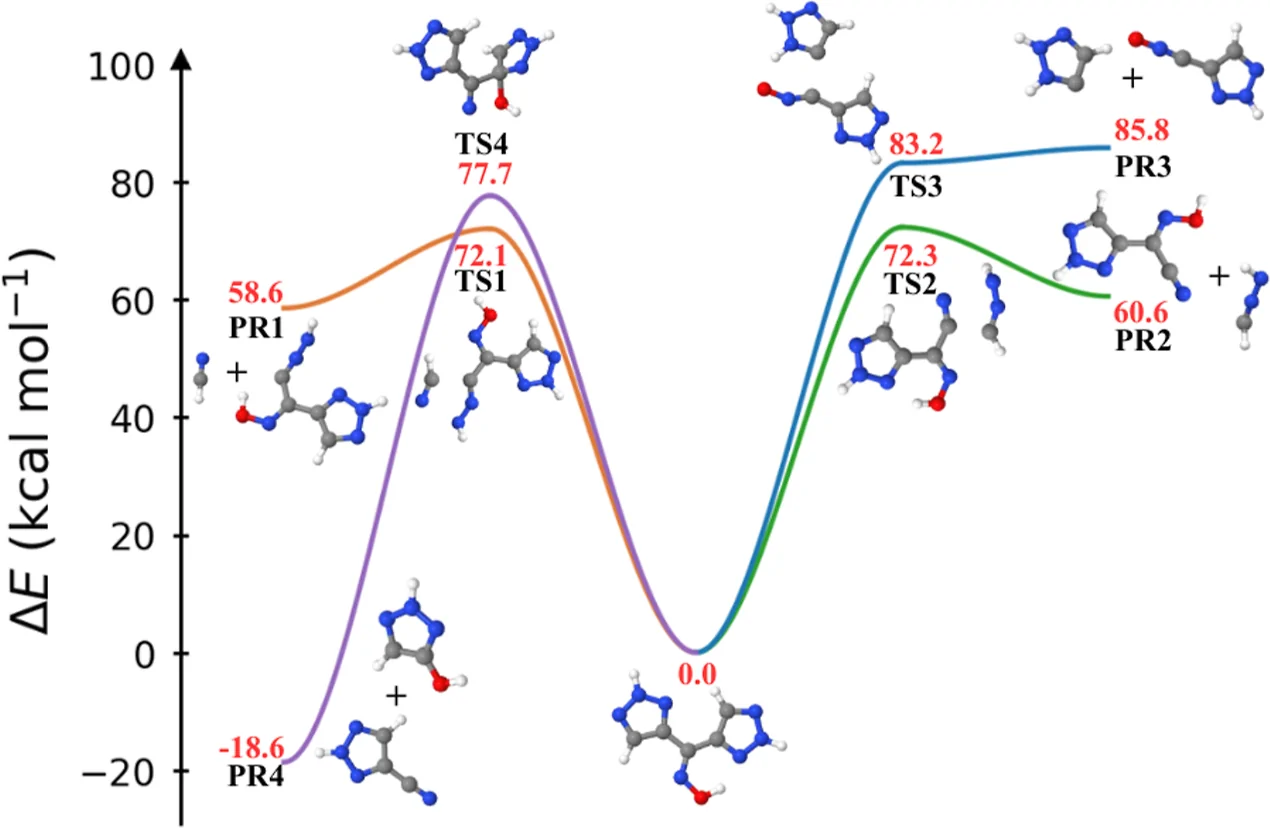
Simplified diagram of the most relevant fragmentation reactions of the Tz2-HA system obtained from the AutoMeKin exploration. Energies, in kcal mol^–1^, are ZPE-corrected DLPNO–CCSD­(T)-F12a reference values relative to the most stable Tz2-HA conformer.

For the four decomposition reactions of the Tz2-HA system, Table 6 summarizes the deviations of the high-level methods considered here from the DLPNO–CCSD­(T)-F12a relative energies. For r^2^SCAN-3c ML-CC, a substantial deviation is found only for TS4 (−7.3 kcal mol^–1^). In contrast, the remaining approaches, and DFT in particular, display significant errors for several stationary points. Notably, the stationary points for which UMA and MACE-OMOL-0 exhibit large deviations are also those showing sizeable discrepancies at the ωB97X-D3/def2-TZVP level. Nevertheless, whereas DFT shows a pronounced error for TS4, both foundation models yield only moderate deviations for this TS. Overall, with the exception of r^2^SCAN-3c ML-CC, the high-level methods tend to overestimate the relative energies. When all reactions obtained with the high-level methods are included in the analysis, the MAEs calculated for the barrier heights, shown in Figure S8 of the SI, confirm the presence of significant deviations. To some extent, these large absolute errors may be associated with the increased size and complexity of the system. Nevertheless, the calculations support the use of the MLIPs considered in this work as practical surrogates for DFT methods.

**Table 6:** Energy Deviations (in kcal mol^–1^) with Respect to DLPNO–CCSD­(T)-F12a for the Stationary Points of the Relevant Channels of the Tz2-HA System^a^

configuration	DFT	UMA	MACE-OMOL-0	r2SCAN-3c ML-CC
TS1	10.13 (82.23)	6.85 (78.95)	6.41 (78.51)	–**0.32** (71.76)
TS2	9.58 (81.88)	6.21 (78.51)	7.36 (79.66)	**0.70** (73.00)
TS3	0.51 (83.71)	**0.17** (83.37)	–1.08 (82.12)	0.25 (83.45)
TS4	6.65 (84.35)	**2.25** (79.95)	2.57 (80.27)	–7.27 (70.43)
PR1	7.83	6.85	6.47	–**0.06**
PR2	7.67	5.42	5.12	**–1.35**
PR3	–2.14	**–0.38**	–0.80	–1.43
PR4	2.38	1.25	0.90	**–0.35**

a The ZPE-corrected energy of the most stable conformer of TZ2-ha was used as the reference zero. Values in parentheses correspond to the TS energies, i.e., the BHs. Bold values indicate the smallest absolute deviation for each configuration.

For each high-level method, RRKM rate coefficients were calculated using Eq. (1). As in the MEA case, the reference RRKM calculations combined CC electronic energies with ωB97X-D3/def2-TZVP vibrational frequencies. For Tz2-HA, the electronic energies correspond to the DLPNO–CCSD­(T)-F12a reference level defined above. We note that, for this system, several modes are predicted to have very low vibrational frequencies (see Table S3). As discussed above, the harmonic treatment of such modes may introduce substantial uncertainties in the calculated densities and sums of states. In addition, a more rigorous treatment of channel 3, and more generally of reactions involving loose transition states, would require a variational RRKM treatment,^38^ since the conventional RRKM approach adopted here places the TS at the saddle point on the PES. Nevertheless, the present kinetic analysis is intended primarily to assess qualitatively how the vibrational frequencies predicted by the different methods may affect the product branching fractions, thereby complementing their evaluation in terms of BHs and REs.

The product population fractions as a function of excitation energy are shown in Figure 8. The reference results, shown as dashed lines, indicate that the decomposition kinetics is dominated by channel 1, whose population fraction is close to 0.9 at the lowest excitation energies investigated. Channel 2 makes a smaller contribution to the overall decomposition, with a product population fraction close to 0.1. Note that the BH of channel 1 is only 0.2 kcal mol^–1^ lower than that of channel 2 (see Figure 7). To a large extent, the dominance of channel 1 over channel 2 arises from the lower values of the low-frequency modes of TS1 compared with those of TS2, as shown in Table S3 of the Supporting Information. Lower transition-state frequencies lead to a larger sum of states, which in turn increases the RRKM rate coefficient (see eq 1). The abundances of the products associated with channels 1 and 2 decrease slightly as the excitation energy increases, leading to a small contribution from channel 3 at high excitation energies. The contribution from channel 4 is negligible over the entire energy range investigated. The larger product fraction of channel 3 relative to channel 4, despite the barrier height of the former being 5.5 kcal mol^–1^ higher, can be explained by the higher values of the low-frequency modes of TS4 compared with those of TS3. These higher frequencies lead to a smaller sum of states for TS4 than for TS3, thereby reducing the rate coefficient associated with channel 4.

**Figure 8:**
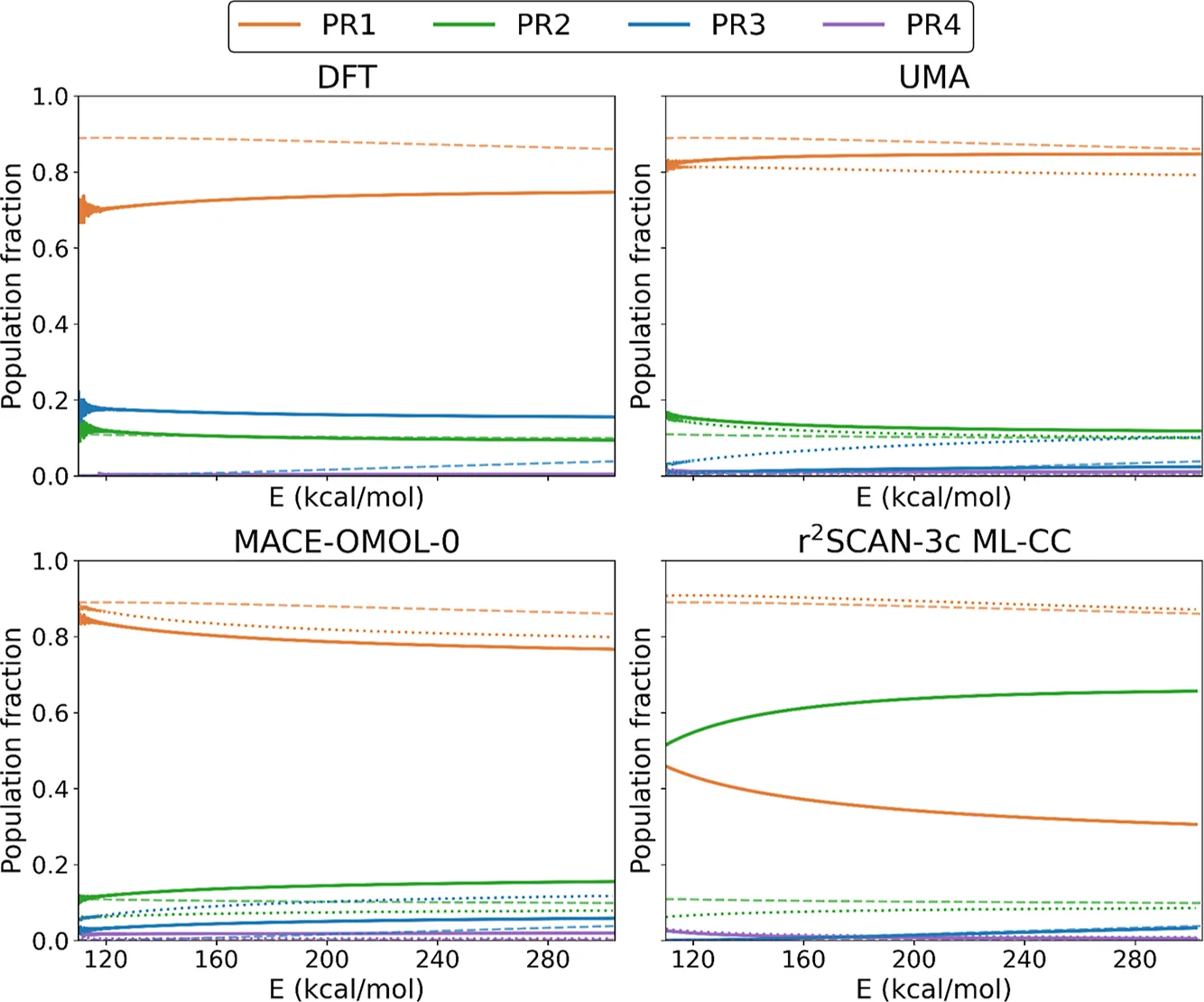
Product population fractions for Tz2-HA decomposition as a function of excitation energy for the competing channels shown in Figure 7. Dashed lines in each panel denote the reference-level populations obtained from DLPNO–CCSD­(T)-F12a/cc-pVDZ-F12//ωB97X-D3/def2-TZVP calculations. For UMA, MACE-OMOL-0, and r^2^SCAN-3c ML-CC, dotted lines denote RRKM populations computed using the ωB97X-D3/def2-TZVP vibrational frequencies.

For the DFT product population fractions, the deviations from the reference values arise solely from differences in the BHs. Although the DFT barrier heights for channels 1 and 2 remain very similar to each other, as shown in Table 6, they differ significantly from the corresponding reference values by comparable amounts. More importantly, the DFT barrier height for channel 3 lies less than 2.0 kcal mol^–1^ above those of channels 1 and 2. Owing to the pronounced loose character of TS3, this TS exhibits the lowest values for the low-frequency modes. Consequently, the contribution of channel 3 to the overall decomposition exceeds that of channel 2.

The product population fractions evaluated with UMA and MACE-OMOL-0 resemble each other. As reported in Table 6, both MLIPs predict similar barrier heights. The small differences between the calculated product fractions arise from slight changes in the barrier heights and vibrational frequencies. When the vibrational frequencies obtained from the ωB97X-D3/def2-TZVP calculations are used, the product population fractions, displayed as dotted lines, become even more similar to each other, as expected.

The results obtained with r^2^SCAN-3c ML-CC show significant disagreement with the reference product fractions. For this method, channel 2 is the dominant channel, even though its BH is 1.2 kcal mol^–1^ higher than that of channel 1. As shown in Table S3 of the SI, the low-frequency modes of TS2 have smaller values than those of TS1. However, when the ωB97X-D3/def2-TZVP frequencies are used, the calculated product fractions become very similar to the reference results. Note that the contribution of channel 4 to the overall fragmentation is negligible, even though this channel has the lowest BH at this level of theory. This behavior arises from the high values of the low-frequency modes of TS4, which exhibits a pronounced tight character. These results indicate that, within the present RRKM analysis, the differences between the r^2^SCAN-3c vibrational frequencies and the ωB97X-D3/def2-TZVP reference frequencies contribute significantly to the deviations observed in the calculated product population fractions. In contrast, the results obtained with the UMA and MACE-OMOL-0 foundation models show better agreement with the reference population fractions. This behavior is consistent with the fact that these MLIPs were trained on energies and gradients computed at a level of theory similar to ωB97X-D3/def2-TZVP.

## 5 Summary and Conclusions

In this work, we have extended the capabilities of AutoMeKin^6^ by presenting a complete integration of ORCA 6 ^8,9^ into the high-level refinement stage of the workflow, together with the integration of the UMA^19^ and MACE-OMOL-0 ^20^ foundation models and Δ-ML corrections.^10^ Compared with the simpler post-processing use of ORCA introduced in our previous work,^10^ the present implementation provides direct access to ORCA within the automated AutoMeKin workflow. Because ORCA 6 is freely available to academic users and offers a broad range of electronic-structure methods, this development increases both the accessibility and flexibility of high-level reaction-network refinement. The new implementation allows reaction networks generated at a low computational level to be refined using conventional DFT or ab initio methods, ML interatomic potentials, or ML-corrected quantum-mechanical approaches within a unified automated framework.

The performance of the new workflow was assessed for five systems: MEA, three cyclobutadiene-derived radicals, cBD–OH, cBD-CN, and cBD-CCH, and the nitrogen-rich Tz2-HA system. These examples were selected to probe different regimes of chemical complexity and training set coverage. MEA represents a favorable case, since related structures are present in both the OMol25 and Green reaction datasets, whereas the cyclobutadiene derivatives and Tz2-HA constitute more challenging out-of-distribution cases for the ML-based approaches. For all systems, the low-level PM7 explorations generated sizable reaction networks, which were subsequently refined using DFT, r^2^SCAN-3c ML-CC, UMA, and MACE-OMOL-0.

For MEA, all high-level approaches reproduced the main unimolecular decomposition channels, and the deviations in BHs and REs relative to the CCSD­(T)-F12a reference values were generally below 2 kcal mol^–1^. The RRKM analysis showed that the product population distribution is governed mainly by barrier heights at low excitation energies, while vibrational frequencies become increasingly important as the excitation energy increases. In particular, the crossing between the acetaldehyde + ammonia and ethenamine + water populations reflects the different growth of the transition-state sums of states associated with the dominant channels.

For the cyclobutadiene-derived radical systems, AutoMeKin recovered the key barrierless association channels reported previously for the reactions of cyclobutadiene with OH, CN, and CCH radicals while also revealing additional fragmentation and isomerization pathways. The comparison with DLPNO–CCSD­(T)-F12a reference energies shows that UMA and MACE-OMOL-0 generally provide energy profiles of comparable quality to the DFT calculations used in this work, even for systems expected to be underrepresented in the OMol25 training set. The r^2^SCAN-3c ML-CC approach often yields small energy deviations, but its practical usefulness is limited by the cost of the underlying electronic-structure calculations and by cases in which the baseline method does not locate relevant channels.

The Tz2-HA system provides a more stringent test because of its size, nitrogen-rich composition, and complex fragmentation pathways. Larger deviations from the CC reference values are observed for several stationary points, particularly for DFT and the MLIPs. Nevertheless, UMA and MACE-OMOL-0 still recover the relevant decomposition channels and provide product population fractions that are in reasonable agreement with the reference RRKM results. The kinetic analysis further highlights the importance of vibrational frequencies in the evaluation of rate coefficients. In particular, differences between r^2^SCAN-3c and ωB97X-D3/def2-TZVP vibrational frequencies contribute significantly to the deviations observed in the corresponding product population fractions, whereas the UMA and MACE-OMOL-0 results show better agreement with the reference populations.

Taken together, the cyclobutadiene-derived radicals and Tz2-HA provide a set of challenging out-of-distribution tests for the MLIPs considered here. For these systems, sizeable deviations from the CC reference values are sometimes observed not only for UMA and MACE-OMOL-0 but also for the ωB97X-D3/def2-TZVP calculations. Since the DFT level used to generate the OMol25 reference data is expected to have an accuracy comparable to that of ωB97X-D3/def2-TZVP, such MLIP errors should not be interpreted solely as evidence of poor transferability. Rather, they also reflect the intrinsic difficulty of the reactive regions explored in this work and the limitations of the underlying electronic-structure descriptions used to generate training data.

Overall, the present results show that MLIPs can be effectively incorporated into automated reaction-discovery workflows and used as practical surrogates for DFT in high-level reaction-network refinement. Although the accuracy of the predicted networks remains sensitive to the optimization algorithm, the electronic-structure model, and the coverage of the training data, the kinetically relevant pathways are generally retained across the different approaches. From a practical perspective, these results suggest a useful strategy for AutoMeKin users: reaction networks may be refined efficiently in the high-level module using UMA or MACE-OMOL-0, followed by high-level electronic-structure single-point calculations, preferably CCSD­(T)-based when feasible, on the stationary points associated with the most relevant channels. This final validation step provides a reliable way to assess the accuracy of the proposed mechanism while retaining most of the computational savings offered by MLIP-based refinement. This strategy has recently been advocated by Kendall and Zádor,^24^ who benchmarked UMA within the KinBot automated kinetics workflow. The present work supports this view in the context of AutoMeKin and extends it to both UMA and MACE-OMOL-0, opening new possibilities for efficient reaction-network explorations in larger and more complex systems.

The good performance of the Δ-ML strategy when combined with an accurate baseline method also suggests a promising direction for future work. In particular, CC single-point energies computed on stationary points optimized with MLIPs could be collected to develop Δ-ML corrections on top of UMA or MACE-OMOL-0, in close analogy with the r^2^SCAN-3c ML-CC model used here. Such ML-corrected foundation potentials could combine the efficiency and broad chemical coverage of universal MLIPs with improved agreement with high-level CC energetics.

The integration of ORCA 6, UMA, MACE-OMOL-0, and Δ-ML corrections therefore substantially increases the flexibility of AutoMeKin and provides a foundation for future developments, including ML-corrected foundation potentials and active-learning strategies for chemically complex reactive systems.

## Supplementary Material



## Data Availability

The newly developed interface integrating the ML models into the AutoMeKin workflow is available from the GitHub repository cited in ref 25. All datasets generated in this study have been uploaded to Zenodo.^58^ The archive contains the reaction-network data, including optimized structures and corresponding energies from the low-level explorations, the high-level refined networks obtained with the four independent levels of theory, the coupled-cluster single-point reference energies, and the main output files of the multiconfigurational calculations.

## Notes

Description of the reaction-matching protocol used to compare reactions across levels of theory; mean absolute errors in barrier heights and reaction enthalpies for the DFT, machine-learning potentials, and r^2^SCAN-3c ML-CC methods relative to the CC reference; case study of the PR3 channel of the cBD-CCH reaction, including the UMA-derived IRC profile and a CASSCF IRC starting from the corresponding TS; and vibrational frequencies used in the RRKM calculations (PDF)

## List of Acronyms

ASE: Atomistic Simulation Environment
BHs: barrier heights
CASSCF: complete active space self-consistent field
CBS: complete basis set limit
CC: coupled cluster
CCSD­(T): coupled cluster singles and doubles with perturbative triples correction
DFT: density functional theory
eMPGNN: equivariant message-passing graph neural network
HF: Hartree-Fock
IRC: intrinsic reaction coordinate
MAE: mean absolute error
MEA: monoethanolamine
MEP: minimum energy path
ML: machine learning
MLIP: machine-learning interatomic potential
NN: neural network
OMol25: Open Molecules 2025 dataset
OOD: out-of-distribution
PES: potential energy surface
QM: quantum mechanical
REs: reaction energies
RMSD: root-mean-square deviation
ROHF: restricted open-shell Hartree-Fock
RRKM: Rice–Ramsberger–Kassel–Marcus theory
RXN: reaction network
SQM: semiempirical quantum mechanical
TS: transition state
UMA: universal models for atoms.

## References

[1] Dewyer A. L., Argüelles A. J., Zimmerman P. M. (2018). Methods for exploring reaction space
in molecular systems. WIREs Comput. Mol. Sci..

[2] Martínez-Núñez, E. In Handbook of Electronic Structure Theory; Hochlaf, M. , Barone, V. , Eds.; Elsevier, 2026; pp 485–500.

[3] Martínez-Núñez E. (2015). An automated
method to find transition states using chemical dynamics simulations. J. Comput. Chem..

[4] Martínez-Núñez E. (2015). An automated
transition state search using classical trajectories initialized at
multiple minima. Phys. Chem. Chem. Phys..

[5] Rodríguez A., Rodríguez-Fernández R., A. Vázquez S., L. Barnes G., J. P. Stewart J., Martínez-Núñez E. (2018). tsscds2018:
A code for automated discovery of chemical reaction mechanisms and
solving the kinetics. J. Comput. Chem..

[6] Martínez-Núñez E., Barnes G. L., Glowacki D. R., Kopec S., Peláez D., Rodríguez A., Rodríguez-Fernández R., Shannon R. J., Stewart J. J. P., Tahoces P. G., Vazquez S. A. (2021). AutoMeKin2021:
an open-source program for automated reaction discovery. J. Comput. Chem..

[7] Martínez-Núñez, E. Publications Using Automekin. https://emartineznunez.github.io/AutoMeKin/docs/works.html, 2026; AutoMeKin Documentation. (accessed June 8, 2026).

[8] Neese F. (2012). The ORCA program
system. WIREs Comput. Mol. Sci..

[9] Neese F. (2025). Software Update:
the ORCA program system version 6.0. WIREs Comput.
Mol. Sci..

[10] Rodríguez-Lopez O., Martínez-Núñez E., Vázquez S. A., Fernández B. (2026). Efficient machine learning barrier
height and reaction
enthalpy corrections for low- and midlevel quantum mechanical calculations. Small Struct..

[11] Frisch, M. J. ; Gaussian 16, Revision C.01; Gaussian Inc: Wallingford CT, 2016.

[12] Manby, F. R. , Miller, T. , Bygrave, P. , Ding, F. , Dresselhaus, T. , Batista-Romero, F. , Buccheri, A. , Bungey, C. , Lee, S. , Meli, R. , , entos: a quantum molecular simulation package. 2019 10.26434/chemrxiv.7762646.v2.

[13] Stewart J. J. P. (2007). Optimization
of parameters for semiempirical methods V: modification of NDDO approximations
and application to 70 elements. J. Mol. Model..

[14] Binkley J. S., Pople J. A., Hehre W. J. (1980). Self-consistent
molecular orbital
methods. 21. Small split-valence basis sets for first-row elements. J. Am. Chem. Soc..

[15] Grimme S., Hansen A., Ehlert S., Mewes M. (2021). r2SCAN-3c: a “swiss
army knife” composite electronic-structure method. J. Chem. Phys..

[16] Grambow C. A., Pattanaik L., Green W. H. (2020). Reactants, products, and transition
states of elementary chemical reactions based on quantum chemistry. Sci. Data.

[17] Spiekermann K. A., Pattanaik L., Green W. H. (2022). Fast predictions of reaction barrier
heights: toward coupled-cluster accuracy. J.
Phys. Chem. A.

[18] Levine D. S. (2025). Open Molecules 2025
(Omol25) Dataset, Evaluations, and Models. arXiv.

[19] Wood, B. M. UMA: A family of universal models for atoms. 2025; https://arxiv.org/abs/2506.23971.

[20] MACE-OMOL model GitHub repository. 2025; https://github.com/ACEsuit/mace-foundations.

[21] Batatia I., Kovacs D. P., Simm G. N. C., Ortner C., Csanyi G. (2022). MACE: Higher
order equivariant message passing neural networks for fast and accurate
force fields. Adv. Neural Inf. Process. Syst..

[22] Young T. A., Johnston-Wood T., Deringer V. L., Duarte F. (2021). A transferable
active-learning
strategy for reactive molecular force fields. Chem. Sci..

[23] Ghorai S., Staub R., Harabuchi Y., Nakano T., Varnek A., Maeda S. (2026). Toward reaction vessel
mimicry: machine learning-assisted automated
exploration of alkene polymerization and its transferability. J. Chem. Theory Comput..

[24] Kendall D. T., Zádor J. (2026). Benchmarking the UMA Foundation interatomic potential
for gas-phase chemical kinetics. J. Phys. Chem.
A.

[25] AutoMeKin-X: Next-Generation Development Version of Automekin. 2026; https://github.com/ComputationalChem-USC/AutoMeKin-X.git, GitHub repository, (accessed 06 08 2026).

[26] Fukui K. (1970). Formulation
of the reaction coordinate. J. Phys. Chem..

[27] Guerrero-Méndez L., Lema-Saavedra A., Jiménez E., Fernández-Ramos A., Martínez-Núñez E. (2023). Gas-phase
formation of glycolonitrile
in the interstellar medium. Phys. Chem. Chem.
Phys..

[28] Hjorth
Larsen A. (2017). The atomic simulation environment a Python library
for working with atoms. J. Phys. Condens. Matter.

[29] Hermes E. D., Sargsyan K., Najm H. N., Zádor J. S. (2022). an open-source
automation-friendly molecular saddle point optimizer. J. Chem. Theory Comput..

[30] Stewart J. J. P. (2013). Optimization
of parameters for semiempirical methods VI: more modifications to
the NDDO approximations and re-optimization of parameters. J. Mol. Model..

[31] Riplinger C., Neese F. (2013). An efficient and near
linear scaling pair natural orbital based local
coupled cluster method. J. Chem. Phys..

[32] Riplinger C., Sandhoefer B., Hansen A., Neese F. (2013). Natural triple excitations
in local coupled cluster calculations with pair natural orbitals. J. Chem. Phys..

[33] Saitow M., Becker U., Riplinger C., Valeev E. F., Neese F. (2017). A new near-linear
scaling, efficient and accurate, open-shell domain-based local pair
natural orbital coupled cluster singles and doubles theory. J. Chem. Phys..

[34] da
Silva G. (2012). Atmospheric chemistry of 2-aminoethanol (MEA): reaction of the NH_2_• CHCH_2_OH radical with O_2_. J. Phys. Chem. A.

[35] Xie H.-B., Li C., He N., Wang C., Zhang S., Chen J. (2014). Atmospheric
chemical reactions of monoethanolamine initiated by OH radical: mechanistic
and kinetic study. Environ. Sci. Technol..

[36] Parks C., Hughes K. J., Pourkashanien M. (2022). Furthering the understanding of product
formation in monoethanolamine degradation: A mechanistic DFT study. Int. J. Greenh. Gas Control..

[37] Alam A., da Silva G. (2024). Oxidation of Aminoacetaldehyde Initiated by the OH
Radical: A Theoretical Mechanistic and Kinetic Study. Atmosphere.

[38] Gilbert, R. G. ; Smith, S. C. Theory of Unimolecular and Recombination Reactions; Blackwell scientific publ.: Oxford, 1990.

[39] Beyer T., Swinehart D. (1973). Algorithm
448: number of multiply-restricted partitions. Commun. ACM.

[40] Ayala P. Y., Schlegel H. B. (1998). Identification and treatment of internal
rotation in
normal mode vibrational analysis. J. Chem. Phys..

[41] Ellingson B. A., Lynch V. A., Mielke S. L., Truhlar D. G. (2006). Statistical thermodynamics
of bond torsional modes: tests of separable, almost-separable, and
improved Pitzer–Gwinn approximations. J. Chem. Phys..

[42] Pitzer K. S., Gwinn W. D. (1942). Energy levels and
thermodynamic functions for molecules
with internal rotation. I. Rigid frame with attached tops. J. Chem. Phys..

[43] Vansteenkiste P., Van Speybroeck V., Marin G. B., Waroquier M. (2007). The 1-D hindered
rotor approximation. Theor. Chem. Acc..

[44] Zheng J., Yu T., Papajak E., Alecu I. M., Mielke S. L., Truhlar D. G. (2011). Practical
Methods for Including Torsional Anharmonicity in Thermochemical Calculations
on Complex Molecules: The Internal-Coordinate Multi-Structural Approximation. Phys. Chem. Chem. Phys..

[45] Zheng J., Truhlar D. G. (2013). Quantum thermochemistry:
multistructural method with
torsional anharmonicity based on a coupled torsional potential. J. Chem. Theory Comput..

[46] Truhlar D.
G., Garrett B. C. (1984). Variational
transition state theory. Annu. Rev. Phys. Chem..

[47] Bao J. L., Truhlar D. G. (2017). Variational transition
state theory: theoretical framework
and recent developments. Chem. Soc. Rev..

[48] Largo M., Sanz-Novo M., Redondo P. (2025). Formation of hydroxy, cyano and ethynyl
derivatives of C_4_H_4_ isomers in the interstellar
medium. Phys. Chem. Chem. Phys..

[49] Mardirossian N., Head-Gordon M. (2016). *ω* B97M-V: a combinatorially
optimized, range-separated hybrid, meta-GGA density functional with
VV10 nonlocal correlation. J. Chem. Phys..

[50] Rappoport D., Furche F. (2010). Property-optimized gaussian basis sets for molecular
response calculations. J. Chem. Phys..

[51] Hellweg A., Rappoport D. (2015). Development of new auxiliary basis functions of the
Karlsruhe segmented contracted basis sets including diffuse basis
functions (def2-SVPD, def2-TZVPPD, and def2-QVPPD) for RI-MP2 and
RI-CC calculations. Phys. Chem. Chem. Phys..

[52] Roos B. O., Taylor P. R., Siegbahn P. E. M. (1980). A complete active space SCF method
(CASSCF) using a density matrix formulated super-CI approach. Chem. Phys..

[53] Dunning T. H. J. (1989). Gaussian
basis sets for use in correlated molecular calculations. I. The atoms
boron through neon and hydrogen. J. Chem. Phys..

[54] Angeli C., Cimiraglia R., Evangelisti S., Leininger T., Malrieu J.-P. (2001). Introduction of *N*-electron valence
states for multireference perturbation theory. J. Chem. Phys..

[55] Angeli C., Cimiraglia R., Malrieu J.-P. (2001). *N*-electron valence
state perturbation theory: A fast implementation of the strongly contracted
variant. Chem. Phys. Lett..

[56] Angeli C., Cimiraglia R., Malrieu J.-P. (2002). *N*-electron valence
state perturbation theory: A spinless formulation and an efficient
implementation of the strongly contracted and of the partially contracted
variants. J. Chem. Phys..

[57] González-Vázquez J., Martínez-Núñez E., Vázquez S., Santamaría J., Bañares L. (2004). RRKM and direct MP2/6-31G­(d, p) quasiclassical
trajectory study of the H2 elimination in the photodissociation of
vinyl chloride at 193 nm. Chem. Phys. Lett..

[58] Rodríguez-López O., Martínez-Núñez E., Vázquez S. A., Fernández B. (2026). Zenodo repository for the chemical reactions databases. Zenodo.

