# Supplementary material for: Enhancing the Efficiency and Flexibility of AutoMeKin: Integrating ORCA and Machine-Learning Potentials
Source: J Chem Theory Comput. 2026 Oct 6;22(20):10816–33. doi: 10.1021/acs.jctc.6c01565 (PMC13718547; doi:10.1021/acs.jctc.6c01565)
Supplement: Supplementary file 1 [file ct6c01565_si_001.pdf]

Supporting information for:

Enhancing the Efficiency and Flexibility of AutoMeKin: Integrating ORCA and Machine-Learning Potentials

Omar Rodríguez-López, Emilio Martínez-Núñez,* Berta Fernández,* Saulo A. Vázquez*

Departamento de Química Física, Universidade de Santiago de Compostela, E-15782 Santiago de Compostela, Spain.

E-mail:

omar.rodriguez.lopez@usc.es,emilio.nunez@usc.es,berta.fernandez@usc.es,saulo.vazquez@usc.es

Contents

1. Reaction matching protocol **S3**

2. Performance of the HL methods **S5**

1. Reaction matching protocol

Reaction matching between the different levels of theory was carried out in two stages (Screening 1 and Screening 2, Figure S1) to ensure that only genuinely equivalent reactions were compared. In the first stage, adjacency matrices were constructed for each TS geometry by assigning a bond between two atoms if their interatomic distance falls within 1.2 times the sum of their covalent radii. These adjacency matrices, labeled by atomic element, were used to verify that candidate pairs share the same bond connectivity pattern, and were further required to have no bond length differing by more than 15% between levels — criteria that together discard pairs whose connectivity is identical but whose geometries differ substantially. The reaction path associated with each surviving candidate was then validated by applying the same connectivity and bond-length criteria to its endpoints (minima and/or products), considering both direct and reverse IRC directions; only candidates for which these criteria were also satisfied at both endpoints were retained as a matched reaction. In the second stage, symmetry-corrected RMSDs were computed for the TS pairs of each matched reaction, and only those with RMSD $\leq$ 0.20 Å were retained as matched TS pairs.

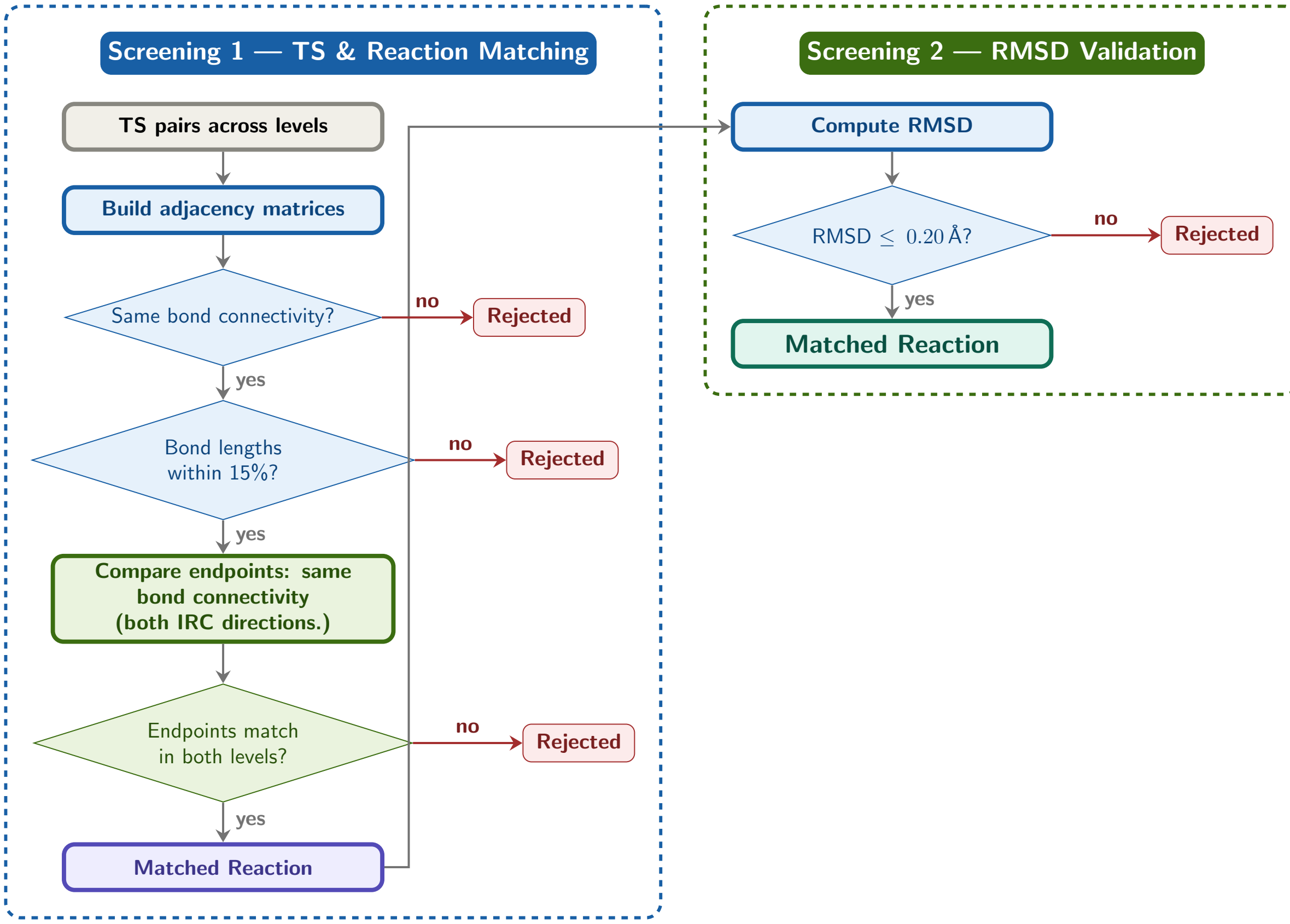

Figure S1: Two-stage workflow used to match transition states (TS) and reactions across levels of theory. In Screening 1, candidate TS pairs are filtered by bond connectivity and bond length agreement, and the corresponding reaction paths are validated by comparing the same criteria at their endpoints in both IRC directions, yielding a set of matched reactions. In Screening 2, symmetry-corrected RMSDs are computed for the TS pairs of each matched reaction, and only pairs below the 0.20 Å threshold are retained as matched TS pairs.

Table S1: Summary of high-level refinement statistics for the five test systems. S-1 and S-2 denote the number of candidates surviving Screening 1 and Screening 2, respectively (see Figure S1).

	MEA		cBD-CN		cBD-CCH		cBD-OH		Tz2-HA	
Levels	S-1	S-2	S-1	S-2	S-1	S-2	S-1	S-2	S-1	S-2
DFT - UMA	268	130	406	174	291	136	93	43	39	20
DFT - MACE	255	76	338	136	262	109	85	33	36	20
DFT - r^2SCAN-3c ML-CC	275	153	385	214	270	150	78	46	37	18
UMA - MACE	296	155	411	216	337	163	126	64	54	41
DFT - DFT(ASE/SELLA)	295	118	-	-	-	-	-	-	-	-

2. Performance of the HL methods

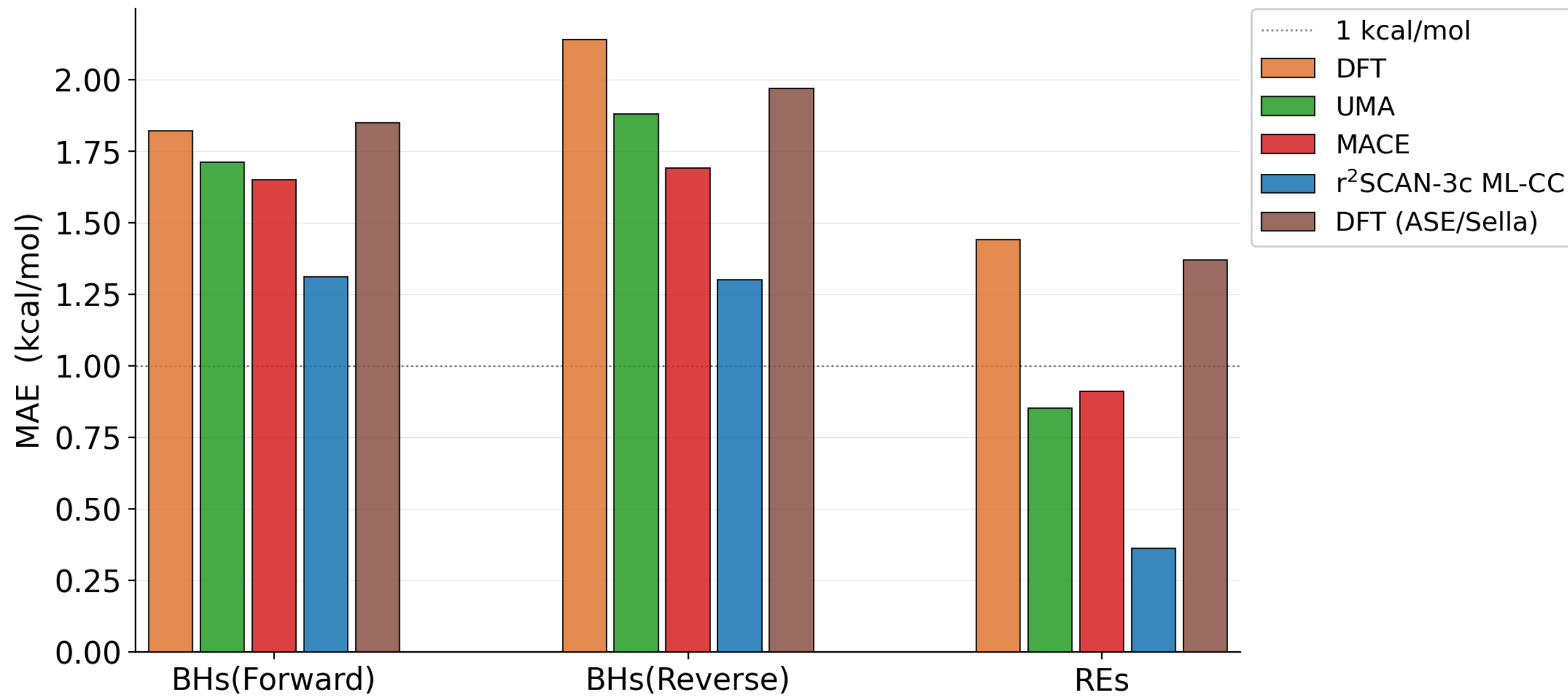

Figure S2: Mean absolute errors for DFT, DFT with the Sella optimizer, UMA, MACE, and r^2SCAN-3c ML-CC relative to CCSD(T)-F12 reference values for the MEA dataset. All reactions were included for DFT, whereas for the other levels of theory, MAEs were calculated only for reactions in the S-2 group.

Table S2: Vibrational frequencies (cm^{-1}) of the TSs for the reaction channels of MEA.

	DFT					UMA					MACE-OMOL-0					r^2SCAN-3c				
TS	TS1	TS2	TS3	TS4	TS5	TS1	TS2	TS3	TS4	TS5	TS1	TS2	TS3	TS4	TS5	TS1	TS2	TS3	TS4	TS5
ν_1	174	174	129	221	106	180	176	123	223	113	155	174	127	225	97	174	175	140	228	88
ν_2	260	285	286	294	158	248	273	288	291	159	228	269	281	288	154	252	280	244	293	113
ν_3	334	397	416	402	293	328	387	414	373	293	337	390	408	373	285	325	409	371	418	257
ν_4	502	444	515	501	341	501	441	511	480	338	490	439	504	459	333	485	444	474	490	348
ν_5	521	501	596	532	547	516	499	590	533	547	509	490	571	510	532	513	484	537	554	512
ν_6	573	589	635	612	692	560	590	606	608	694	567	587	594	591	672	561	571	598	629	651
ν_7	675	734	741	683	725	660	730	730	679	721	662	718	726	665	721	654	739	664	677	720
ν_8	880	791	806	748	838	869	786	807	759	841	872	781	804	750	833	853	786	751	742	742
ν_9	1001	823	979	951	1014	998	814	969	944	1020	1001	814	946	939	991	979	830	965	927	966
ν_{10}	1106	944	1060	961	1088	1096	937	1049	948	1085	1085	929	1039	944	1058	1077	936	1037	954	1052
ν_{11}	1138	1029	1126	1051	1110	1126	1025	1114	1034	1104	1131	1018	1100	1023	1097	1087	998	1076	984	1067
ν_{12}	1168	1134	1187	1172	1146	1159	1121	1168	1150	1129	1155	1115	1161	1129	1113	1127	1111	1140	1180	1122
ν_{13}	1232	1158	1214	1206	1190	1227	1152	1201	1190	1174	1219	1151	1191	1187	1166	1205	1123	1197	1202	1167
ν_{14}	1291	1222	1241	1265	1247	1277	1210	1223	1239	1232	1276	1207	1214	1221	1217	1256	1218	1218	1287	1208
ν_{15}	1347	1266	1344	1380	1352	1330	1255	1326	1361	1342	1320	1247	1315	1349	1331	1336	1256	1357	1367	1358
ν_{16}	1436	1401	1416	1396	1390	1420	1376	1399	1377	1372	1412	1363	1385	1368	1366	1422	1382	1396	1387	1399
ν_{17}	1534	1447	1455	1452	1450	1505	1420	1433	1430	1437	1498	1414	1426	1419	1421	1503	1438	1465	1426	1470
ν_{18}	1639	1528	1519	1472	1481	1628	1509	1502	1457	1470	1637	1499	1486	1452	1453	1621	1503	1511	1458	1473
ν_{19}	1680	1639	1676	1643	1676	1666	1649	1659	1627	1657	1669	1610	1647	1614	1655	1659	1571	1662	1595	1663
ν_{20}	1959	1684	2244	1666	2278	1917	1668	2189	1643	2194	1923	1662	2175	1631	2136	1914	1670	2135	1648	2176
ν_{21}	2897	3114	3061	2293	3088	2902	3099	3056	2314	3078	2887	3100	3051	2360	3034	2819	3051	3014	2219	2995
ν_{22}	3152	3162	3129	3065	3094	3147	3158	3122	3063	3082	3127	3152	3115	3056	3057	3099	3128	3062	3014	3052
ν_{23}	3271	3262	3152	3117	3201	3265	3256	3150	3110	3189	3236	3241	3126	3103	3178	3225	3235	3102	3072	3167
ν_{24}	3362	3549	3428	3563	3586	3337	3521	3368	3538	3551	3326	3501	3371	3487	3538	3324	3488	3365	3498	3531
ν_{25}	3592	3626	3577	3711	3674	3556	3596	3551	3678	3636	3525	3575	3521	3654	3621	3557	3578	3522	3665	3631
ν_{26}	3669	3824	3668	3846	3876	3631	3774	3643	3791	3828	3598	3735	3612	3788	3804	3645	3764	3624	3772	3799

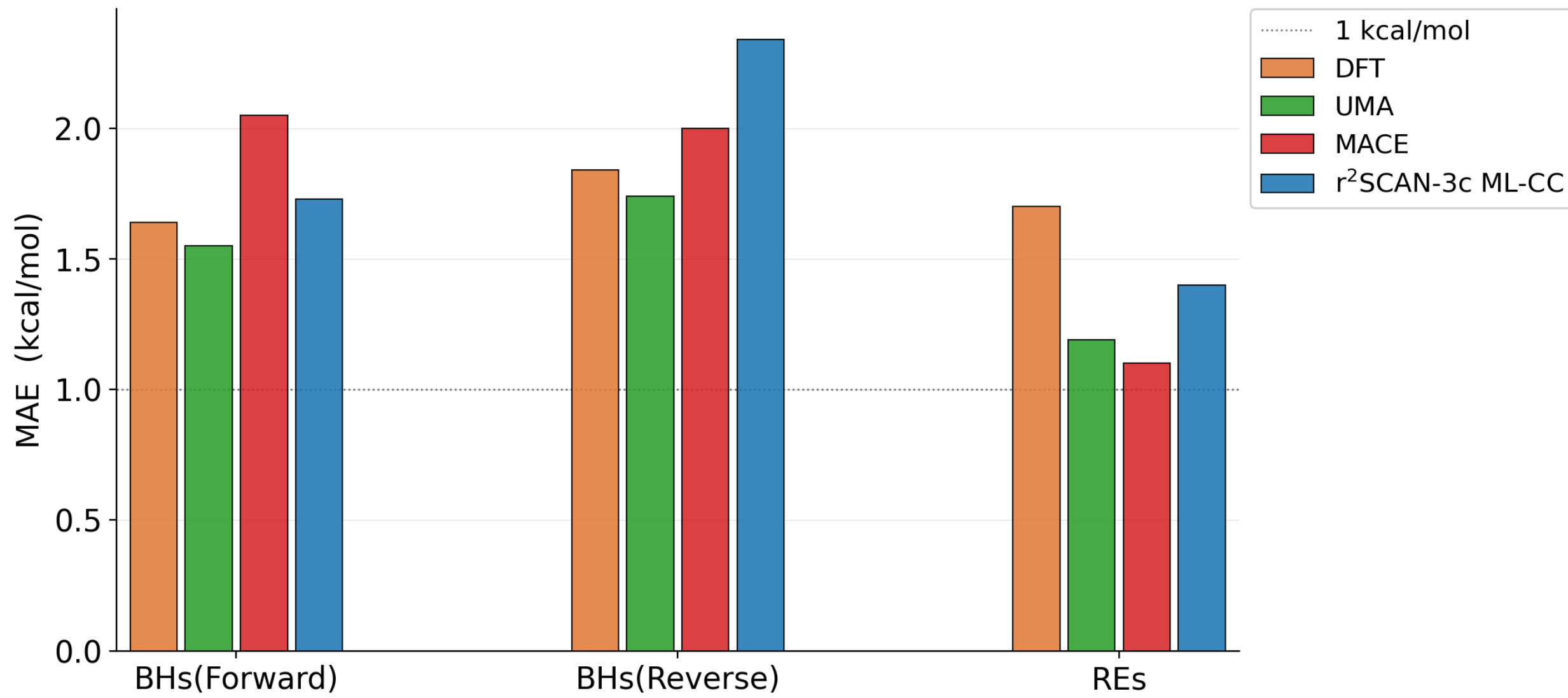

Figure S3: Mean absolute errors for DFT, UMA, MACE, and r^{2}SCAN-3c ML-CC relative to the DLPNO-CCSD(T)-F12 reference for the cBD-OH dataset. All reactions were included for DFT, whereas for the other levels of theory, MAEs were calculated only for reactions in the S-2 group.

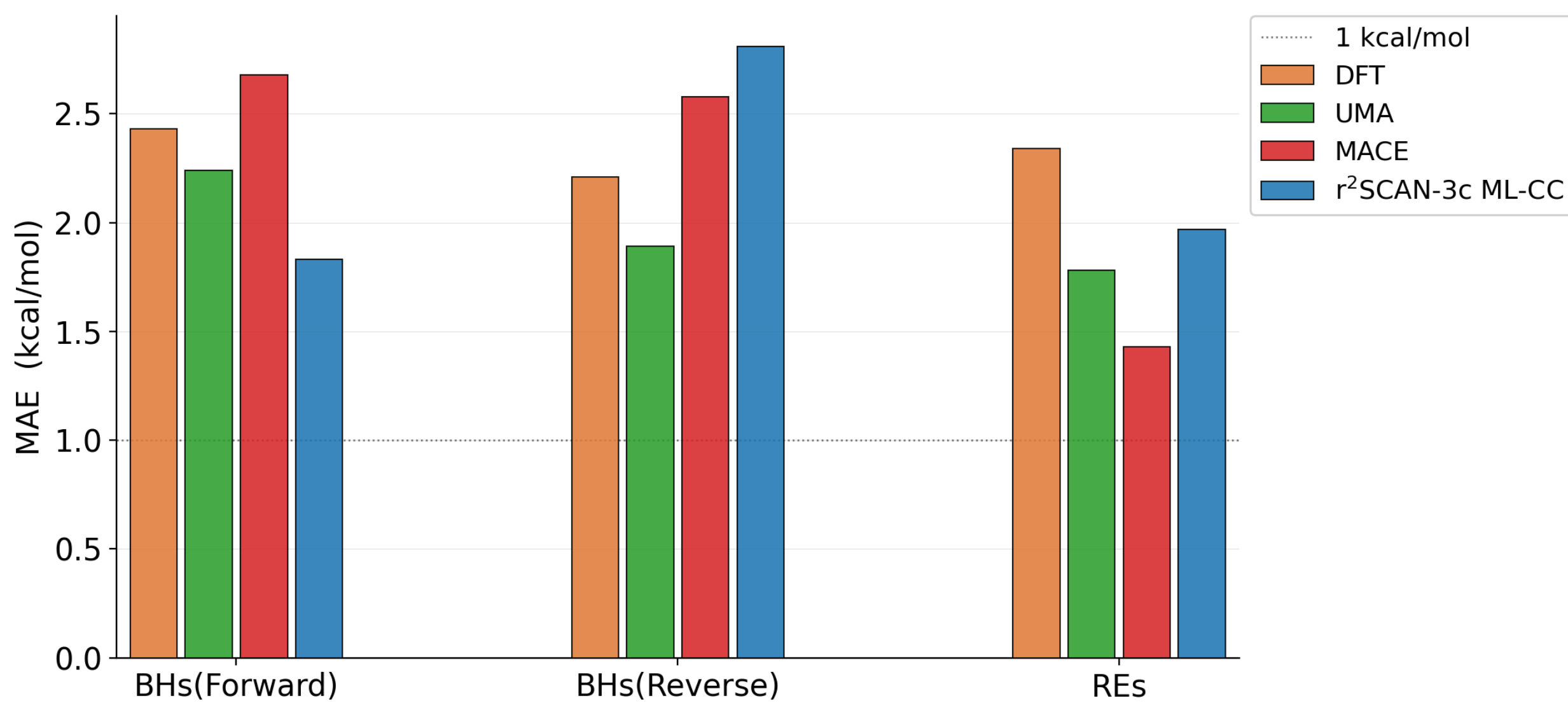

Figure S4: Mean absolute errors for DFT, UMA, MACE, and r^{2}SCAN-3c ML-CC relative to the DLPNO-CCSD(T)-F12 reference for the cBD-CN dataset. All reactions were included for DFT, whereas for the other levels of theory, MAEs were calculated only for reactions in the S-2 group.

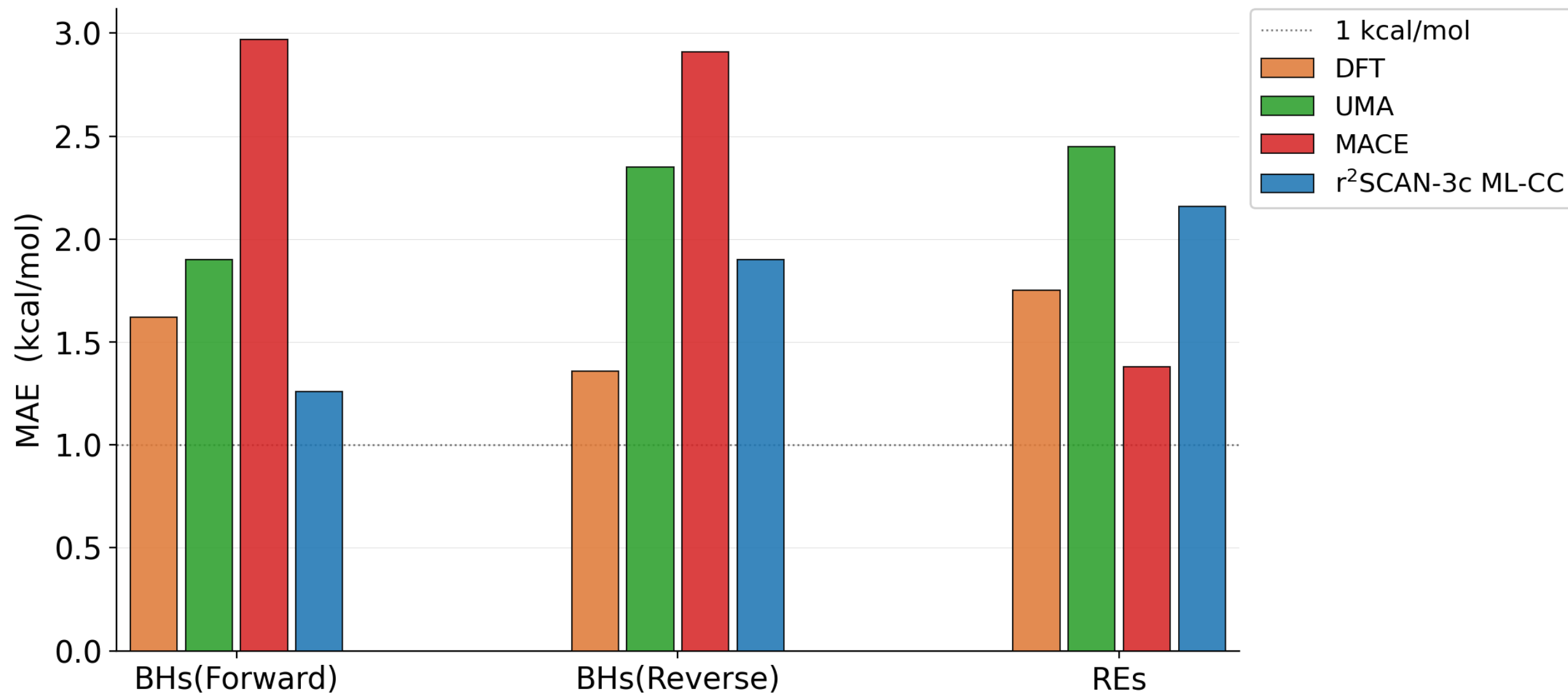

Figure S5: Mean absolute errors for DFT, UMA, MACE, and r^{2}SCAN-3c ML-CC relative to the DLPNO-CCSD(T)-F12 reference for the cBD-CCH dataset. All reactions were included for DFT, whereas for the other levels of theory, MAEs were calculated only for reactions in the S-2 group.

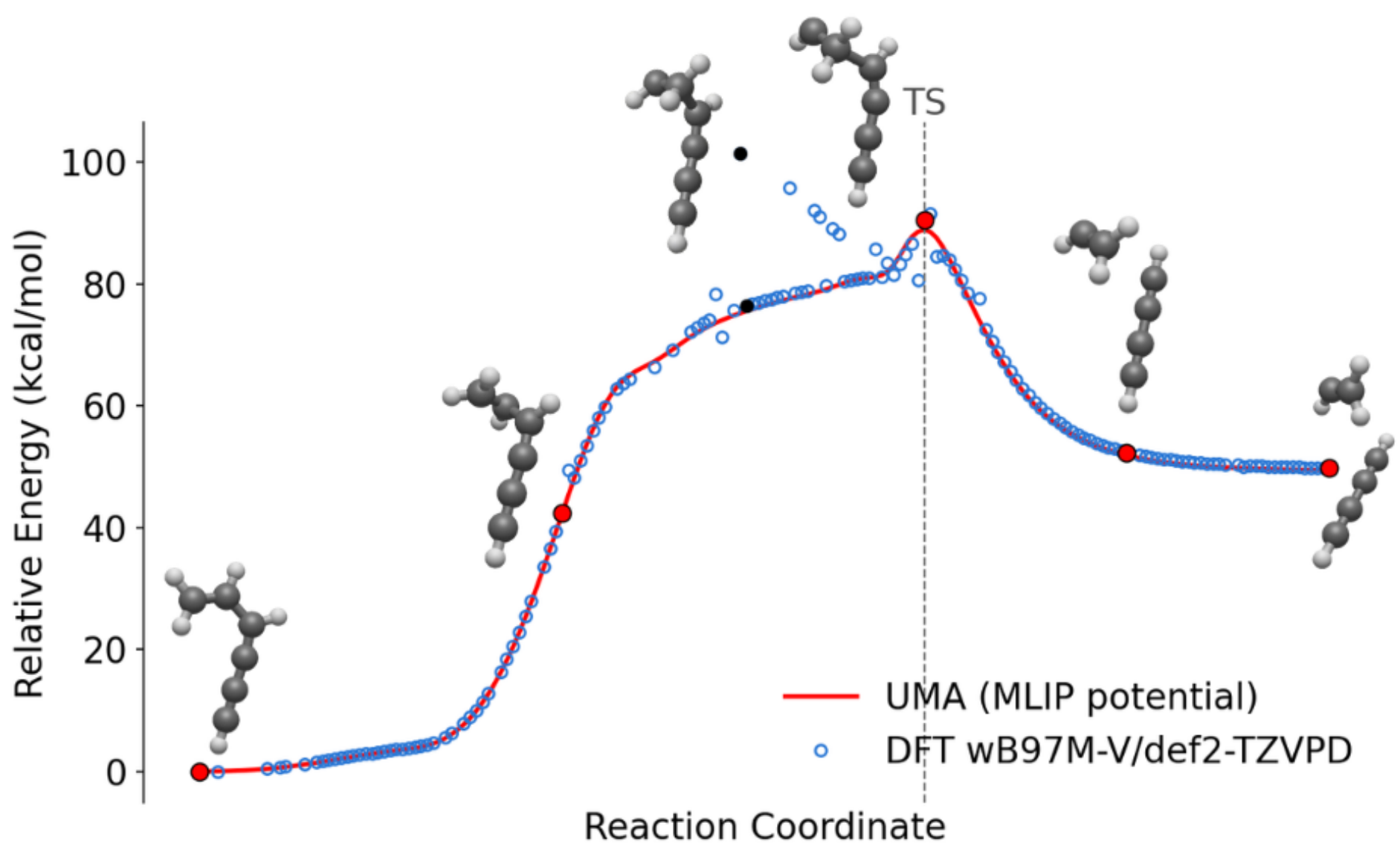

Figure S6: Intrinsic reaction coordinate energy profile for the MIN3 $\rightarrow$ PR3 reaction in the c-C_4H_4 + CCH reaction system, compared with single-point ωB97M-V/def2-TZVPD energies. Gaps in the DFT curve correspond to points where the SCF procedure failed to converge.

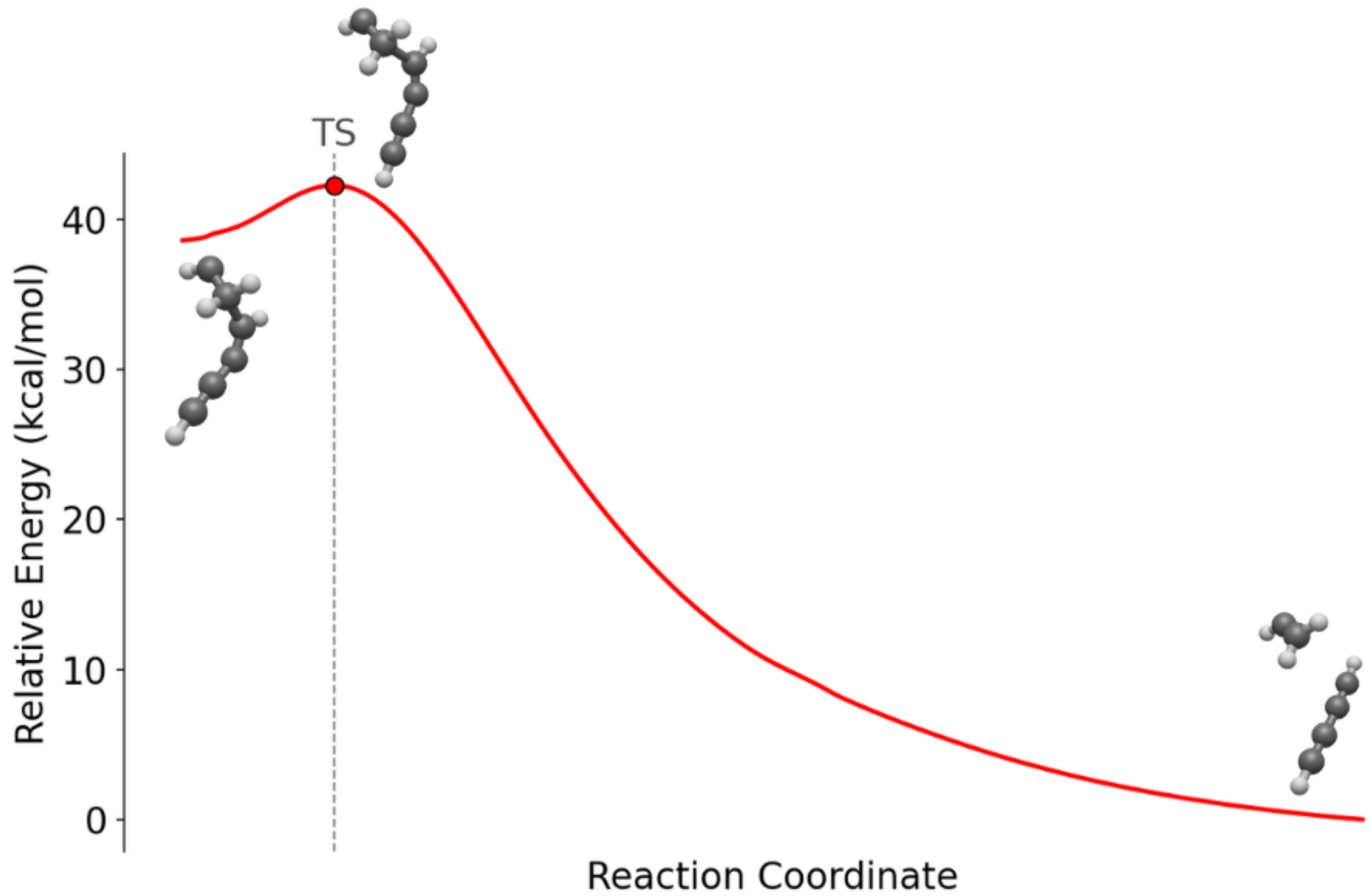

Figure S7: Intrinsic reaction coordinate calculated at the CASSCF(11,11) level, starting from the transition-state structure analogous to TS6 predicted by UMA.

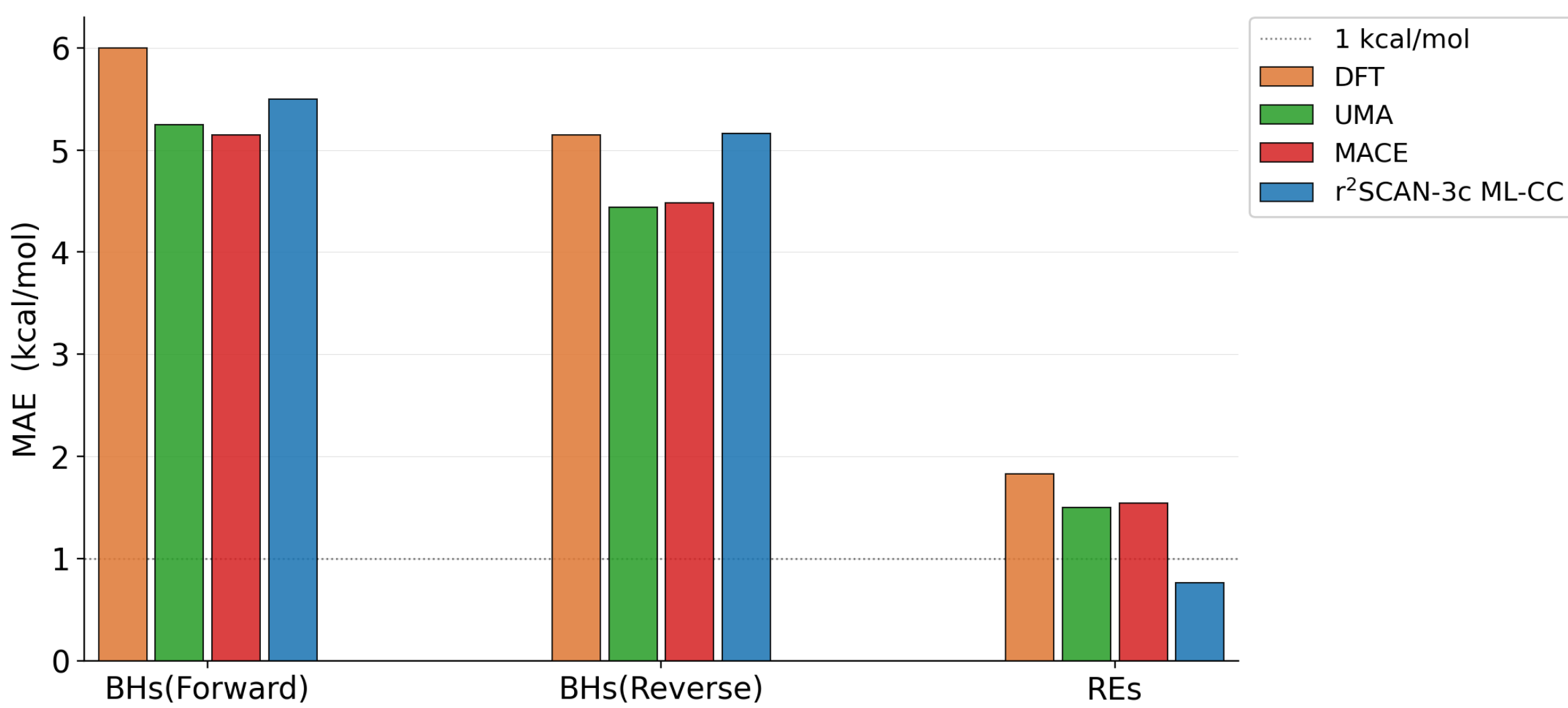

Figure S8: Mean absolute errors for DFT, UMA, MACE, and r^{2}SCAN-3c ML-CC relative to the DLPNO-CCSD(T)-F12 reference for the Tz2-HA dataset. All reactions were included for DFT, whereas for the other levels of theory, MAEs were calculated only for reactions in the S-2 group.

Table S3: Vibrational frequencies (cm^{-1}) of the TSs for the reaction channels of Tz2-HA (part 1 of 2).

	DFT				UMA				MACE-OMOL-0				r^2SCAN-3c			
TS	TS1	TS2	TS3	TS4	TS1	TS2	TS3	TS4	TS1	TS2	TS3	TS4	TS1	TS2	TS3	TS4
ν_1	20	38	4	33	16	36	19	37	22	32	12	34	29	34	14	44
ν_2	32	67	65	80	37	66	64	73	37	65	65	69	43	43	47	79
ν_3	84	93	75	88	84	93	80	85	93	92	77	81	87	68	60	86
ν_4	100	124	89	168	100	126	89	169	96	123	92	156	118	118	84	165
ν_5	156	197	150	209	164	197	146	186	171	191	144	191	172	144	134	198
ν_6	210	203	186	228	208	209	189	214	212	205	190	210	208	163	158	223
ν_7	237	222	213	304	243	235	212	249	249	242	222	278	216	181	168	316
ν_8	276	286	329	344	282	282	320	331	271	278	345	310	245	210	234	329
ν_9	316	301	362	391	316	298	358	373	314	299	355	366	297	317	351	374
ν_{10}	342	359	401	424	337	352	392	390	343	369	397	412	328	330	399	390
ν_{11}	432	375	463	452	433	370	450	439	436	378	452	427	372	354	455	426
ν_{12}	457	484	534	559	452	475	528	564	448	479	525	540	450	434	470	561
ν_{13}	464	501	553	609	465	499	548	603	477	498	555	563	467	437	533	577
ν_{14}	501	536	597	641	498	526	590	626	506	524	584	632	479	462	560	618
ν_{15}	555	584	670	653	554	569	662	648	547	565	660	639	554	494	644	649
ν_{16}	571	591	705	688	566	587	700	678	579	596	698	672	560	527	692	665
ν_{17}	629	629	723	735	614	625	715	712	606	621	710	699	579	537	701	709
ν_{18}	637	634	732	739	632	628	725	729	637	627	724	730	627	608	712	718
ν_{19}	665	674	896	758	660	656	885	750	656	653	882	755	653	664	776	742
ν_{20}	683	721	901	815	675	715	891	824	693	712	887	829	694	716	856	779
ν_{21}	740	730	932	892	732	721	936	883	731	720	948	878	722	734	861	865
ν_{22}	778	772	958	910	765	765	953	895	766	761	955	883	745	782	942	896
ν_{23}	788	826	993	962	778	806	987	954	816	812	986	889	757	826	977	935
ν_{24}	896	892	1006	984	893	872	998	980	885	899	998	980	868	863	987	965

Table S3: Vibrational frequencies (cm^{-1}) of the TSs for the reaction channels of Tz2-HA (part 2 of 2).

	DFT				UMA				MACE-OMOL-0				r^2SCAN-3c			
TS	TS1	TS2	TS3	TS4	TS1	TS2	TS3	TS4	TS1	TS2	TS3	TS4	TS1	TS2	TS3	TS4
ν_{25}	916	933	1028	1010	904	921	1019	988	904	914	1017	989	873	892	1004	973
ν_{26}	1007	1007	1122	1020	1000	999	1111	1004	999	1000	1113	1008	979	953	1012	991
ν_{27}	1027	1045	1132	1060	1018	1037	1113	1057	1019	1036	1113	1056	994	991	1111	1042
ν_{28}	1077	1131	1156	1092	1052	1104	1147	1101	1048	1102	1150	1134	1015	1024	1114	1098
ν_{29}	1122	1160	1209	1176	1113	1145	1191	1167	1113	1143	1189	1172	1079	1094	1171	1136
ν_{30}	1203	1211	1221	1192	1191	1199	1206	1176	1190	1198	1199	1193	1155	1153	1186	1147
ν_{31}	1247	1253	1267	1241	1245	1237	1249	1201	1248	1237	1246	1231	1222	1210	1204	1198
ν_{32}	1272	1271	1281	1262	1261	1266	1263	1229	1261	1268	1263	1243	1226	1234	1225	1202
ν_{33}	1293	1292	1282	1273	1277	1276	1274	1251	1277	1275	1276	1268	1238	1240	1239	1220
ν_{34}	1374	1357	1381	1290	1364	1339	1370	1271	1364	1340	1365	1275	1315	1320	1325	1226
ν_{35}	1412	1417	1399	1377	1400	1407	1390	1357	1400	1404	1387	1367	1369	1347	1343	1314
ν_{36}	1440	1440	1423	1405	1425	1430	1411	1396	1424	1429	1412	1393	1391	1388	1354	1349
ν_{37}	1481	1492	1441	1425	1464	1479	1427	1408	1462	1475	1420	1410	1445	1393	1376	1368
ν_{38}	1539	1573	1497	1457	1523	1550	1485	1416	1522	1545	1489	1436	1490	1499	1436	1394
ν_{39}	1576	1591	1525	1489	1568	1583	1512	1482	1569	1580	1512	1483	1525	1548	1495	1459
ν_{40}	1723	1675	1575	1540	1723	1676	1569	1522	1718	1671	1567	1542	1611	1639	1515	1498
ν_{41}	2003	2028	1589	1588	2006	2008	1579	1581	1971	2019	1580	1582	1945	1965	1539	1538
ν_{42}	2039	2108	2184	1833	2026	2120	2197	1797	1995	2071	2210	1719	1977	2069	2139	1727
ν_{43}	3332	3305	3049	3292	3332	3295	3023	3281	3321	3304	2916	3277	3302	3252	3250	3258
ν_{44}	3336	3324	3284	3293	3337	3329	3280	3290	3338	3322	3254	3283	3307	3268	3256	3261
ν_{45}	3480	3480	3318	3685	3444	3435	3314	3658	3410	3429	3302	3659	3402	3415	3283	3627
ν_{46}	3689	3512	3692	3715	3661	3492	3661	3685	3657	3486	3649	3687	3631	3642	3616	3653
ν_{47}	3878	3694	3693	3725	3827	3668	3668	3712	3832	3661	3660	3716	3797	3800	3639	3681
